# Prion-specific and surrogate CSF biomarkers in Creutzfeldt-Jakob disease: diagnostic accuracy in relation to molecular subtypes and analysis of neuropathological correlates of p-tau and Aβ42 levels

**DOI:** 10.1007/s00401-017-1683-0

**Published:** 2017-02-15

**Authors:** Francesca Lattanzio, Samir Abu-Rumeileh, Alessia Franceschini, Hideaki Kai, Giulia Amore, Ilaria Poggiolini, Marcello Rossi, Simone Baiardi, Lynne McGuire, Anna Ladogana, Maurizio Pocchiari, Alison Green, Sabina Capellari, Piero Parchi

**Affiliations:** 1 Department of Biomedical and Neuromotor Sciences, University of Bologna, Bologna, Italy; 2 IRCCS, Institute of Neurological Sciences, Bologna, Italy; 3 National CJD Research and Surveillance Unit, University of Edinburgh, Edinburgh, Scotland, UK; 4 Department of Neurosciences, Istituto Superiore di Sanità, Rome, Italy; 5 Department of Neurological Sciences, Tohoku University Graduate School of Medicine, Sendai, Japan

**Keywords:** Human prions, Biomarkers, RT-QuIC, Amyloid-beta, Tauopathy, Alzheimer’s disease, Protease-sensitive prionopathy

## Abstract

The differential diagnosis of Creutzfeldt-Jakob disease (CJD) from other, sometimes treatable, neurological disorders is challenging, owing to the wide phenotypic heterogeneity of the disease. Real-time quaking-induced prion conversion (RT-QuIC) is a novel ultrasensitive in vitro assay, which, at variance with surrogate neurodegenerative biomarker assays, specifically targets the pathological prion protein (PrP^Sc^). In the studies conducted to date in CJD, cerebrospinal fluid (CSF) RT-QuIC showed good diagnostic sensitivity (82–96%) and virtually full specificity. In the present study, we investigated the diagnostic value of both prion RT-QuIC and surrogate protein markers in a large patient population with suspected CJD and then evaluated the influence on CSF findings of the CJD type, and the associated amyloid-β (Aβ) and tau neuropathology. RT-QuIC showed an overall diagnostic sensitivity of 82.1% and a specificity of 99.4%. However, sensitivity was lower in CJD types linked to abnormal prion protein (PrP^Sc^) type 2 (VV2, MV2K and MM2C) than in typical CJD (MM1). Among surrogate proteins markers (14-3-3, total (t)-tau, and t-tau/phosphorylated (p)-tau ratio) t-tau performed best in terms of both specificity and sensitivity for all sCJD types. Sporadic CJD VV2 and MV2K types demonstrated higher CSF levels of p-tau when compared to other sCJD types and this positively correlated with the amount of tiny tau deposits in brain areas showing spongiform change. CJD patients showed moderately reduced median Aβ42 CSF levels, with 38% of cases having significantly decreased protein levels in the absence of Aβ brain deposits. Our results: (1) support the use of both RT-QuIC and t-tau assays as first line laboratory investigations for the clinical diagnosis of CJD; (2) demonstrate a secondary tauopathy in CJD subtypes VV2 and MV2K, correlating with increased p-tau levels in the CSF and (3) provide novel insight into the issue of the accuracy of CSF p-tau and Aβ42 as markers of brain tauopathy and β-amyloidosis.

**Electronic supplementary material:**

The online version of this article (doi:10.1007/s00401-017-1683-0) contains supplementary material, which is available to authorized users.

## Introduction

Human prion diseases, also known as transmissible spongiform encephalopathies (TSE), are rapidly progressive neurodegenerative disorders caused by prion protein misfolding [57]. With an annual incidence of ~2 cases per million persons sporadic Creutzfeldt-Jakob disease (sCJD) is by far the most common form (85–90% of cases) [53], followed by genetic CJD (gCJD) and fatal familial insomnia (10–15% of cases), which are linked to point or insertion mutations in the prion protein gene (*PRNP*) [14]. Rare acquired forms of CJD caused by human to human or animal to human transmission of infectious prions [74], and atypical disease variants with a slower progression, such as Gerstmann-Sträussler-Scheinker syndrome (GSS), prion protein-cerebral amyloid angiopathy (PrP-CAA) and variably protease-sensitive prionopathy (VPSPr) complete the phenotypic spectrum of human prion diseases of the CNS [25, 81].

CJD is increasingly recognised in the differential diagnosis of rapidly progressive neurological syndromes, highlighting the need for reliable tools to provide an early clinical diagnosis [23, 33, 61, 62]. The ageing population, the improved awareness of CJD as a heterogeneous disorder covering a wider phenotypic spectrum than previously recognised [53], and the identification of potentially treatable disorders that manifest as rapidly progressive dementia (RPD) [8, 16, 24] have all contributed to this scenario.

Current diagnostic criteria for sCJD [80] were mainly developed for epidemiological purposes and do not take into account the extent of clinical heterogeneity shown by sCJD patients. This is a major cause of the difficulties in the clinical diagnosis and differentiation of prion diseases against other neurological disorders. Currently, at least six major subtypes of sCJD are recognised, which are largely determined by the genotype at the polymorphic codon 129 (encoding methionine, M or valine, V) in *PRNP*, and by the type (type 1 or type 2) of PrP^Sc^ accumulating in the brain [50, 52]. The number and variety of clinical symptoms (especially at onset), the rate of disease progression, and the underlying regional brain pathology vary significantly among sCJD subtypes; this affects the relative accuracy of the proposed diagnostic criteria.

CSF protein assays combined with special MRI techniques such as FLAIR and DWI currently represent the most useful in vivo markers for sCJD [13, 78]. Specifically, several brain-derived CSF proteins serving as surrogate markers for neuronal damage have been considered, alone or in combination, for their utility in supporting the clinical diagnosis of probable CJD. Among them, CSF protein assays for 14‑3-3, t-tau and p-tau (for the calculation of the t-tau/p-tau ratio) have contributed the most promising and significant results regarding sensitivity and specificity in distinguishing CJD from other RPDs, although with a significant heterogeneity both in terms of number of studies and agreement regarding the relative performance of each assay [6, 9, 12, 17, 18, 22, 30, 44, 48, 60, 63, 67, 68, 72, 79]. More recently, the development of the RT-QuIC assay [5], an in vitro fluorimetric assay which is able to indirectly detect very low amounts of prions, based on the capacity of PrP^Sc^ to induce recombinant (rec-) PrP conversion and aggregation, has provided a very promising tool to improve the early diagnosis of human prion diseases in a noteworthy manner given its high degree of sensitivity (82–97%) and specificity (99–100%) [5, 19, 41, 45, 46, 55, 64].

The evaluation of biomarker accuracy for an early clinical diagnosis should be ideally conducted on a clinically based cohort of patients with RPD in which the clinical suspicion of prion disease was raised. Nevertheless, only a few studies considered this approach [6, 9, 17, 48, 62, 71, 80]. Furthermore, only a portion of previous studies specifically analysed the effect of the disease subtype on the sensitivity and specificity of the available biomarker assays [15, 28, 30, 35, 38, 48, 63, 77]. Finally, knowledge about the reliability of measuring CSF proteins such as p-tau and Aβ42 to predict the underlying neuropathology is based on a limited number of studies that often used post-mortem ventricular CSF or brain biopsies to maintain the time lapse between CSF and brain assessment at minimum [11, 65, 69, 70]. In this respect, brains affected by CJD provide the unique opportunity to correlate the CSF findings with the post-mortem neuropathology within a short time interval.

In the present study, we aimed to investigate the utility of several CSF biomarkers (14-3-3, t-tau, p-tau, Aβ42 and rec-PrP seeded conversion by RT-QuIC) in the differential diagnosis of CJD from other neurological disorders, in a large non-selected clinical population suspected to be affected by a prion disease. Furthermore, we assessed the diagnostic accuracy (specificity and sensitivity) of each assay employed in the present study, also considering the molecular subtypes in sporadic cases and the mutation type in genetic CJD patients. Finally, in definite CJD cases we correlated the CSF findings concerning p-tau and Aβ42 with the type and amount of tau and Aβ pathology in the brain.

## Materials and methods

### Inclusion criteria and case classification

We analyzed CSF samples from 1062 patients presenting with a progressive neurological syndrome which prompted the inclusion of CJD in the differential diagnosis at time of lumbar puncture (LP). Samples were from consecutive cases submitted for diagnostic purposes between January 2003 and June 2016, and were analyzed at the Laboratory of Neuropathology (NP-Lab) of the Institute of Neurological Sciences of Bologna (ISNB), a major reference laboratory for prion disease in Italy.

A clinical history of current and past illnesses, periodically updated up to the time of the last data analysis (November 2016), as well as the results of EEG and brain MRI studies, were acquired for each patient. Follow-up clinical data were obtained with the combined effort of neurologists at the ISNB and those at the National CJD Surveillance Unit in Rome. Based on the available clinical, laboratory (EEG and MRI) and neuropathological data, patients were classified at the time of data analysis in diagnostic categories according to the updated WHO criteria for the diagnosis of CJD and related disorders [80] with a modification concerning the categories “possible” (see also below) and “probable” CJD, mainly because no CSF biomarker data were used for case classification. Specifically, four major groups were considered for the data analysis: “*definite*” CJD (*n* = 233), which included all prion positive cases at post-mortem examination (186 sCJD + 17 gCJD +1 VPSPr) and the genetic cases carrying a pathogenic *PRNP* mutation who had no autopsy (*n* = 29), “*probable*” CJD (*n* = 97), consisting of patients fulfilling the clinical criteria for possible CJD and showing either a positive EEG or a positive MRI or both, “*possible*” CJD (*n* = 29), comprising patients in which the primary clinical diagnosis after follow-up remained CJD despite the lack of a positive EEG or MRI, and the “*non*-*CJD*” (*n* = 703). The latter included: (1) 586 patients in whom an alternative diagnosis to prion disease was given, either by post-mortem neuropathological examination (*n* = 81) or by clinical criteria (*n* = 505), and (2) 117 patients not fulfilling the clinical criteria for possible CJD but lacking an alternative diagnosis. Among them 38 showed significant clinical improvement at follow-up, 41 suffered from cognitive decline or disturbances of vigilance without associated neurological signs, 24 had a total disease duration longer than 2 years, while for the remaining 14 cases clinical information were too scanty to reach a reliable classification. However, none of these 14 cases showed a positive CSF assay (14-3-3, t-tau, or RT-QuIC).

Finally, a subgroup of “definite” non-CJD cases (*n* = 212), including those that were prion negative at post-mortem examination (*n* = 81), those showing a clinical evolution incompatible with a prion disease (e.g., improvement or stabilization at follow-up) (*n* = 61, including 21 cases with a clinical diagnosis of encephalitis and 2 of metabolic encephalopathy), and those with an alternative “definitive” clinical diagnosis (e.g., strongly supported by genetic, neuroradiological and/or laboratory findings) (*n* = 70), was also considered for a more accurate calculation of the specificity of the diagnostic tests. Specifically, the group with a “definitive” clinical diagnosis included patients with: (1) a neurodegenerative disease (*n* = 7), either carrying a pathogenic mutation (1 Alzheimer’s disease (AD), 2 fronto-temporal dementia (FTD), 1 Lewy body dementia and 2 Huntington’s disease) or with a clinico-neuroradiological diagnosis (1 superficial siderosis of CNS), (2) a clinico-neuroradiological diagnosis of stroke (*n* = 7), (3) a paraneoplastic syndrome confirmed by the finding of a systemic tumor and/or the presence of anti-(onco)neuronal antibodies in serum and CSF (*n* = 20), (4) a laboratory proven (presence of membrane-associated antineuronal antibodies in CSF) non-paraneoplastic autoimmune encephalitis (*n* = 9), (5) a laboratory confirmed diagnosis of an infectious encephalitis (*n* = 8), with a clinico-neuroradiological diagnosis of CNS malignancy (*n* = 8), with a clinico-neuroradiological diagnosis of metabolic encephalopathy (*n* = 4), with a psychiatric diagnosis (*n* = 4) and with other rare diseases (*n* = 3).

The clinical diagnosis of AD was made according to the 2011 National Institute on Aging and the Alzheimer’s Association workgroup guidelines [43]. In particular, after a clinical follow-up of at least 24 months, all 101 patients with a clinical diagnosis of AD fulfilled criteria for probable AD dementia with high or intermediate evidence of the AD patho-physiological process.

The study was conducted according to the revised Declaration of Helsinki and Good Clinical Practice guidelines. Informed consent was given by study participants or their next of kin.

### Molecular genetic analysis

To identify cases carrying mutations and to determine the genotype at the polymorphic codon 129 of the *PRNP* gene, we carried out a molecular analysis in all subjects with a definite, probable or possible diagnosis of prion disease (*n* = 359), as previously described [32]. Furthermore, all cases with a positive familial history for dementia and those with a clinical history compatible with early onset neurodegenerative dementia (<60 years) were also screened for variants in 22 dementia-associated genes, using the Illumina MiSeq sequencer with the amplicon-based assay TruSeq Custom Amplicon v1.5 (TSCA, Illumina), as described by Beck et al. [7]. Major screened genes included *PSEN1*, *PSEN2*, *APP*, *PRNP*, *GRN*, *MAPT*, and *FUS*.

### CSF biochemical analysis

CSF samples were collected by LP following a standard procedure, centrifuged at 1000×*g* for 10 min and stored in polypropylene tubes at −80 °C until analysis.

#### 14-3-3 protein detection

For this assay, which was performed in all samples (*n* = 1062), 10 µl of CSF were mixed with loading buffer, containing 4 mM EDTA, 6% (w/v) sodium dodecyl sulphate (SDS), 20% glycerol (w/v) and 50 mM Tris–HCl (pH 6.8), heated for 5 min at 100 °C. Proteins were then separated by SDS-PAGE on a 13% gel and transferred to a polyvinylidene fluoride (PVDF) membrane. After blotting, the PVDF membrane was blocked for 60 min with 10% (w/v) non-fat dry milk powder in Tris-buffered saline with 0.1% Tween-20, and incubated overnight at 4 °C with pan-anti-14-3-3 rabbit polyclonal primary antibody sc-629 (1:200, Santa Cruz Biotechnology, Inc). The membrane was then incubated for 1 h at room temperature with an anti-rabbit horseradish peroxidase-linked secondary immunoglobulin diluted 1:3000. The immunoreactive signal was detected by enhanced chemiluminescence on an LAS 3000 camera. Western blot signals were measured by densitometry using AIDA software.

Two CSF controls (with a weak or a strong 14-3-3 signal, respectively) were loaded in duplicate on every gel together with the CSF samples. The immunoreactivity signals were rated as negative, ambiguous or positive, on the basis of the optical densitometric (OD) comparison with the weakly positive control. In particular, the 14-3-3 signal was classified as negative when the 14-3-3 band OD was lower than the control, ambiguous (or weakly positive) when the 14-3-3 OD was up to two times higher than the control, and positive when it was at least two times higher than the control. This decision point was chosen after having analysed the test predictive value at different densitometry value ranges. To maintain a consistent cut-off value determined by the OD of the weakly positive control throughout the whole study, we pooled several CSF samples, aliquoted and stored them at −80 °C until analysis. Furthermore, a systematic comparison of densitometry values between control samples was performed each time a new pooled control sample was introduced, which happened four times during the study.

#### T-tau, p-tau (181P) and Aβ42 protein quantification

These three proteins were quantitatively analysed using commercially available kits based on a sandwich ELISA method, according to the manufacturer’s instructions (INNOTEST, Innogenetics, Gent, Belgium). While the CSF concentration of t-tau was measured in all patients (*n* = 1062), p-tau and Aβ42 assays were performed in subgroups of, respectively, 605 (294 CJD and 311 non-CJD, including 85 AD) (see also Suppl. Table 1) and 339 (208 CJD and 131 non-CJD, including 71 AD) cases. For Aβ42, however, the actual number of samples used for group comparison and neuropathological correlates of CSF protein level was lower (see also paragraph on effect of storage time and Suppl. Table 1). Based on receiver operating characteristic (ROC) curve analysis, the cut-off value chosen for t-tau was 1250 pg/ml, whereas a p-tau level >60 pg/ml and an Aβ42 level <450 pg/ml were considered abnormal based on internal normative values. Specifically, at 1250 pg/ml t-tau reached an area under the curve (AUC) of 0.949 ± 0.07. The optimal cut-off value was chosen after analysing the distribution of sensitivity and specificity at different decision points and calculated as 1250 pg/ml based on maximum potential effectiveness (Youden index, 0.78). At this decision point, the sensitivity and specificity were, respectively, 89.4 and 88.1%. The between-assay coefficients of variation for the t-tau, p-tau and Aβ42 tests were, respectively, 10.0, 9.1 and 13.0%, as determined by internal control samples during the study period. The laboratory performing the analyses participates to the Alzheimer’s Association quality control program on CSF biomarkers [40].

#### PrP^Sc^ detection by RT-QuIC

This assay was performed in a subgroup of 700 samples including 179 definite sCJD, 1 VPSPr, 46 gCJD, 97 probable CJD, 29 possible CJD, and 348 non-CJD control patients affected by other neurological disorders (Table 1).

**Table 1:** Comparison of diagnostic accuracy of CSF biomarkers

Diagnostic categories	14-3-3			t-tau			RT-QuIC		
	*n* positive/*n* total	Sens. (%)	Spec. (%)	*n* positive*/*n* total	Sens. (%)	Spec. (%)	*n* positive/*n* total	Sens. (%)	Spec. (%)
Definite sCJD	155/186	83.3		164/186	88.2		148/179	82.7	
Probable sCJD	80/97	82.5		90/97	92.8		77/97	79.4	
Possible sCJD	24/29	82.8		24/29	82.8		22/29	75.9	
Definite VPSPr	1/1			1/1			0/1		
Codon 129									
MM	172/195	88.2		176/195	90.3		160/190	84.2	
MV	42/72	58.3		60/72	83.3		52/72	72.2	
VV	46/46	100		46/46	100		35/44	79.5	
Genetic CJD	38/46	82.6		42/46	91.3		42/46	91.3	
Mutation type									
E200K-129M	11/16	68.7		13/16	81.2		16/16	100	
V210I-129M	20/21	95.2		21/21	100		20/21	95.2	
E200K-129V	4/4	100		4/4	100		4/4	100	
4-inserts-129M	1/1			1/1			1/1		
D178N-129V	1/2			1/2			0/2		
R208H-129V	0/1			1/1			0/1		
V203I-129M	1/1			1/1			1/1		
All CJD**	298/359	83.0		321/359	89.4		289/352	82.1	
Definite CJD	194/233	83.3		207/233	88.8		190/225	84.4	
All non-CJD	118/703		83.3	84/703		88.1	2/348		99.4
“Definite” non-CJD	79/212		62.7	54/212		74.5	1/163		99.4

* T-tau >1250 pg/ml

** Includes definite, probable, possible and genetic CJD cases

CSF samples were analyzed by the RT-QuIC assay as previously described [41], with minor modifications. Briefly, the RT-QuIC reaction mix contained 10 mM phosphate buffer at pH 7.4, 300 mM NaCl, 1 mM EDTA at pH 8.0, 10 μM thioflavin-T (ThT) and 0.1 mg/ml of Syrian hamster recombinant full-length prion protein (Ha rPrP 23-231, supplied by Bristol Institute of Blood Sciences, Bristol, UK) [41]. All the reaction solutions were freshly prepared and filtered before use with 0.22 µm sterile filters. For this assay, 15 µl of each CSF sample was added in the dark to 85 µl of reaction mix in black clear-bottom 96-well micro plates. Samples were tested in quadruplicate together with a positive (definite CJD) and a negative (non-CJD) control. After sealing, the plate was incubated in a FLUOstar OPTIMA reader at 42 °C, over a period of 120 h with intermittent cycles of shaking (60 s, 700 rpm, double-orbital) and rest (60 s). The fluorescence intensity of ThT-PrP^Sc^ aggregates, expressed as relative fluorescence units (rfu), was taken every 45 min using 450 ± 10 nm (excitation) and 480 ± 10 nm (emission) wavelengths, with a bottom read. A CSF sample was considered prion positive if the mean of at least two out four sample replicates gave a fluorescence signal higher than the threshold cut-off value of 7000 rfu. This threshold represents the mean rfu values of negative samples plus at least five standard deviations. Samples were considered negative if none of the replicates surpassed the chosen cut-off. In case only one replicate went over the threshold, the test was considered ambiguous/unclear and repeated.

### Analysis of the effect of CSF storage time

In the present study, the results of CSF proteins 14-3-3 and t-tau assays have been analyzed prospectively. Samples were sent for diagnostic purposes and the results obtained within a 2–3 week time frame for both assays. At variance, p-tau, Aβ42 and RT-QuIC assays were implemented in the lab at later times (2011 for p-tau and Aβ42, 2013 for the RT-QuIC). As a consequence, samples were analysed in these assays at various times after collection. To determine whether the length of storage had an effect on the results of these assays, we compared the results obtained between groups of samples with increasing storage times. While we found that the length of storage has no effect on p-tau and RT-QuIC (see Suppl. Table 2), a significant effect (significantly lower protein levels) was seen on Aβ42 in the group with the longest storage time (>5 years). We, therefore, limited the analysis of the results of the Aβ42 assay to the samples with a storage time up to 5 years (*n* = 285, 164 CJD and 121 non-CJD).

### Neuropathological analysis

According to a standardized protocol which is used nationwide for prion suspected cases, the right half of the brain is immediately frozen, stored at −80 °C, while the other half (left) is fixed in formalin. Once received by the reference lab (NP-Lab at ISNB), the frozen cerebral and cerebellar hemispheres are cut in coronal sections, and then both the frozen and fixed halves are regionally sampled according to standardized procedures.

Histopathological examination was performed on 7 μm thick sections of formalin-fixed and paraffin-embedded brain tissue blocks. Sections were systematically taken from neo-cortical areas (two for each lobe), limbic cortices (cingulate and insular cortices), basal ganglia (anterior and posterior), thalamus (anterior and posterior), hippocampus (anterior and posterior), amygdala and basal forebrain, midbrain, pons, medulla oblongata and cerebellum (vermis and hemisphere with and without dentate nucleus). Tissues (PrP^Sc^ positive at Western blotting) were processed after decontamination for 1 h in concentrated formic acid (98%). For screening, haematoxylin–eosin stain was performed on all sections according to a standard procedure. Evaluation of spongiform change and immunohistochemical PrP deposits was carried out in all cases of neuropathologically confirmed prion disease on sections from, respectively, 23 and 10 brain regions. The monoclonal antibody 3F4 (1:400, Signet Labs, MA, USA) was used for PrP immunohistochemistry, as described [50], whereas the antibodies 4G8 (1.5000, Signet Labs, MA, USA) and AT8 (1:100, Innogenetics, Gent, Belgium) were used to assess, respectively, Aβ and p-tau immunoreactivity. Neuropathological diagnostic assessments were done by one experienced neuropathologist (PP) in virtually all cases (278 of 284 brains).

#### Assessment of amyloid-beta brain deposits in CJD

For the correlation between CSF Aβ42 concentration and Aβ tissue deposits, brains (*n* = 118) were examined independently by two evaluators (PP and HK) for the extent and topographic progression of Aβ pathology (Thal phases) as described [3]. The median time interval between lumbar puncture and death in this group was 2 months (interquartile range (IQR) 1–4.4). To take into account the variability of Aβ load within brains with the same Thal phase, as well as the contribution of CAA, we carried out a semiquantitative assessment of brain Aβ load within each of the examined brain regions (frontal, temporal, parietal and occipital lobes, amygdala, striatum, hippocampus (CA1 region), midbrain and cerebellum). Parenchymal Aβ pathology was graded (0–7) as follows: 0, entirely negative; 2, rare/sparse deposits; 4, moderate number of deposits; 6, multiple deposits, disseminated. An additional point was added to the total score if core-plaques were also noted. CAA was graded (0–3) as follows: 0, entirely negative; 1, up to two vessels focally involved; 3, more than half of vessels involved or significant involvement of capillaries; 2, intermediate between 1 and 3. CAA was assessed in leptomeninges and parenchyma of all hemispheric lobes, amygdala, striatum, hippocampus/parahippocampal gyrus, midbrain and cerebellum. For each case a cumulative score (0–90) of total semi-quantitatively assessed Aβ load in the brain (parenchymal + angiopathy) was calculated.

#### Semi-quantitative evaluation of primary age-related (PART)/AD-related tau pathology

For the assessment of PART/AD-related pathology, 159 brains (158 CJD and 1 VPSPr) were examined independently by two evaluators (PP and HK) for the extent and topographic progression of tau pathology (Braak stages) [10, 20] as described [2], with some modifications. Specifically, p-tau immunoreactivity was evaluated semi-quantitatively (0-no immunoreactivity; 1-mild; 2-moderate; 3-prominent immunoreactivity) in the following regions: CA1 region of the hippocampus, transentorhinal cortex, entorhinal cortex, parahippocampal gyrus at the level of anterior hippocampus, middle temporal gyrus, middle frontal gyrus, inferior parietal lobule, and occipital cortex (including the calcarine cortex). We (HK and PP) scored the neuronal (cell body), the fine neuritic (threads) tau deposits and the thick neuritis that are part of neuritic plaques, separately. A combined total score (0–24) was given to each case.

#### Assessment of prion-related tau pathology

To search for tau pathology specifically related to CJD, we evaluated p-tau immunoreactivity in brain regions showing the most prominent spongiform changes in brains with no or only minimal AD pathology (*n* = 52). To this aim, we excluded all cases with an AD-related tau score above 10 or a Braak stage higher than II, and evaluated p-tau immunoreactivity in the cerebral cortex, anterior striatum, thalamus, and cerebellum. Likewise, with the AD-related score, a combined score was given after a semi-quantitative assessment (0-no immunoreactivity; 1-mild; 2-moderate; 3-prominent immunoreactivity).

#### Assessment of aging-related tau astrogliopathy (ARTAG)

ARTAG was overall defined and assessed according to Kovacs et al. [37]. However, a detailed topographical examination of astrocytic tau pathology was only carried out in the VPSPr brain, whereas in the CJD brains (*n* = 164) the analysis was limited to the screening phase, using sections of posterior hippocampus and pons.

#### PrP^Sc^ typing

PrP^Sc^ typing was performed in virtually all autopsied CJD cases (197/203; 180 sCJD, 16 gCJD and 1 VPSPr), using brain homogenates from at least four different brain regions (temporal, parietal, occipital cortices, and thalamus) as described [51, 52].

### Prion disease classification

All but 6 (brain examined in a general pathology laboratory) sporadic cases with a definitive diagnosis were given a histotype classification according to the criteria proposed by Parchi et al. [52, 54], which are based on histopathological features, PrP^Sc^ type, and codon 129 genotype. Mixed sCJD types were merged with the “corresponding” pure type based on similarities in the clinical phenotype. Accordingly, the pairs MM(V)1/MM(V)1+2C (from now on abbreviated in the manuscript as MM(V)1), MM2C/MM2C+1 (abbreviated as MM2C), and MV2K/MV2K+2C (abbreviated as MV2K) were merged into three individual groups.

Genetic prion cases were classified according to the type of mutation and the genotype at codon 129 in the mutated allele (and the PrP^Sc^ type when available), while the probable sCJD cases were divided into three groups based on the codon 129 genotype (MM, MV and VV).

### Statistical analyses

CSF levels of t-tau, p-tau, the t-tau/p-tau ratio, Aβ42 and RT-QuIC relative fluorescence responses were analysed using the SPSS software package (version 20). Depending on the data distribution, the Mann–Whitney U test or the Chi-Square test were used to test differences between two groups, while the Kruskal–Wallis or one-way ANOVA (followed by Tukey’s post hoc test) were applied for multiple group comparisons. A Bonferroni correction was applied to multiple comparisons. Data are expressed as median with IQR. The diagnostic utility of each biomarker was evaluated by estimating the sensitivity and specificity. ROC curve analysis was performed to establish the diagnostic accuracy of t-tau and the t-tau/p-tau ratio. The optimal cut-off value for t-tau was chosen using the Youden index. The Youden index for a cut-off is defined by its sensitivity + specificity-1. As the distributions of values were not Gaussian, the Spearman bivariate test was used to detect the strength of correlation between the pathology scores and CSF levels of Aβ42 and p-tau. Values of *p* < 0.05 were considered statistically significant.

Statistical analyses of the effect of age, sex, timing of lumbar puncture, and disease duration on the CSF biomarkers were limited to the largest phenotypically homogeneous CJD group (e.g., MM1) when the parameter was also influenced by the CJD subtype (see also “Results” section). For example, since it is well established that the CJD type has a profound effect on disease duration, the effect of this variable can only reliably be tested within a single CJD subtype.

## Results

### CSF 14-3-3

At the chosen decision point (i.e., protein band immunoreactivity at least two times higher than that of the weakly positive control), 39.2% of tested samples had a positive 14-3-3 protein assay, including 83.3% of definite sCJD, 82.6% of definite gCJD, 82.5% of probable sCJD and 82.8% of possible sCJD cases (Table 1). The overall calculated test sensitivity was 83.0% and the test specificity was 83.3%, while the test positive predictive value (PPV) and negative predictive value (NPV) were, respectively, 71.6 and 90.5%. When the analysis was limited to the definite sCJD and the “definite” non-CJD groups the sensitivity did not change significantly (83.3%), whereas the specificity dropped to 62.7% (Table 1).

When the test sensitivity was examined in sCJD according to the genotype at codon 129 of the *PRNP* gene, patients homozygous for valine (VV) showed the highest sensitivity (100%), while those carrying the heterozygous methionine/valine genotype (MV) showed the lowest (58.3%) (Table 1). The assay sensitivity varied even more significantly among sCJD subtypes, ranging from 0 to 100%. It was highest and full in VV2 (100%), relatively high in MM(V)1 (92.8%), but low in MV2K (50.0%), very low in MM2C (30.0%), and inconsistent in MM2T (0%) (Table 2).

**Table 2:** Sensitivity of CSF biomarkers according to sCJD subtype

Molecular subtypes of definite sCJD	14-3-3		t-tau			RT-QuIC	
	*n* positive/*n* total	Sensitivity (%)		*n* positive/*n* total	Sensitivity (%)	*n* positive/*n* total	Sensitivity (%)
MM(V)1*	103/111	92.8		103/111	92.8	95/107	88.8
VV2	29/29	100		29/29	100	21/27	77.8
MV2K**	13/26	50		20/26	76.9	21/26	80.8
MM2C***	3/10	30		4/10	40	4/9	44.4
Other rare sporadic CJD/TSE types							
MM2T	0/3	0		1/3	33.3	1/3	33.3
VV1	1/1	100		1/1	100	1/1	100
VPSPr (VV)	1/1	100		1/1	100	0/1	0

* The group includes 39 MM1+2C cases

** The group includes 1 atypical MV2K, and 1 MV2K+2C

*** The group includes 4 MM2C+1, and 1 MV2C case

In the gCJD group, the test sensitivity was higher in subjects carrying the V210I-129M haplotype (95.2%) than in those carrying E200K-129M (68.7%) (Table 1).

In the non-CJD control group, a 14-3-3 “false” positive result mainly occurred in patients with subacute neurological pathologies, such as encephalitis or other inflammatory conditions, including paraneoplastic syndromes, or CNS malignancy, and, to a lesser extent, in cases affected by vascular dementia/stroke or neurodegenerative dementias such as AD or Lewy body dementia (Table 3; Suppl. Table 3).

**Table 3:** Influence of diagnostic category on false positive results: 14-3-3 vs t-tau

Diagnostic categories of non-CJD patients	14-3-3 positive/total (%)	t-tau positive/total (%)	Definitive diagnosis (*n* = 212)	
			Path.	Clin.
Alzheimer’s disease	8/101 (7.9)	16/101 (15.8)	15	1
Lewy body dementia	4/72 (5.6)	5/72 (6.9)	13	1
Frontotemporal dementia	3/40 (7.5)	3/40 (7.5)	0	2
Other NDG diseases	1/51 (2.0)	0/51 (0)	1	3
Mixed dementia	0/35 (0)	0/35 (0)	1	0
Vascular dementia/stroke	19/85 (22.4)	15/85 (17.6)	11	7
CNS malignancy	11/18 (61.1)	5/18 (27.8)	8	8
Encephalitis/paraneoplastic syndromes	52/130 (40.0)	24/130 (18.5)	14	58^a^
Toxic/metabolic encephalopathy	2/20 (10.0)	1/20 (5.0)	4	6^b^
Psychiatric disease	0/8 (0)	0/8 (0)	0	4
Other diseases	0/13 (0)	0/13 (0)	1	3
Cause unknown	18/130 (13.8)	15/130 (11.5)	13	38^c^

*NDG* neurodegenerative

^a^21/58 patients improved at follow-up

^b^2 patients improved at follow-up

^c^All these patients improved at follow-up

### CSF total tau

The results of t-tau assay for the different diagnostic groups are summarised in the Table 1. In particular, 38.1% of tested samples showed t-tau levels above the chosen threshold of 1250 pg/ml, including 88.2% of definite sCJD, 91.3% of definite gCJD, 92.8% of probable sCJD, and 82.8% of possible CJD cases (Table 1). The overall calculated test sensitivity was 89.4% and the specificity was 88.1%, while the PPV and NPV were, respectively, 79.3 and 94.2%. As for 14-3-3, the sensitivity value did not change significantly when the analysis was limited to the definite sCJD and the “definite” non-CJD groups, whereas the specificity decreased to 74.5% (Table 1).

As with 14-3-3, the t-tau assay showed a markedly different sensitivity in sCJD based on the codon 129 genotype, the VV genotype being associated with the highest sensitivity (100%), and the MV genotype with the lowest (83.3%). Among the sCJD subtypes, the diagnostic sensitivity was 100% in VV2, 92.8% in MM(V)1, 76.9% in MV2K, 40.0% in MM2C and 33.3% in MM2T (Table 2). Finally, in genetic CJD, the assay sensitivity was higher in patients carrying the V210I-129M haplotype (100%) than in those carrying E200K-129M (81.2%).

**Fig. 1:**
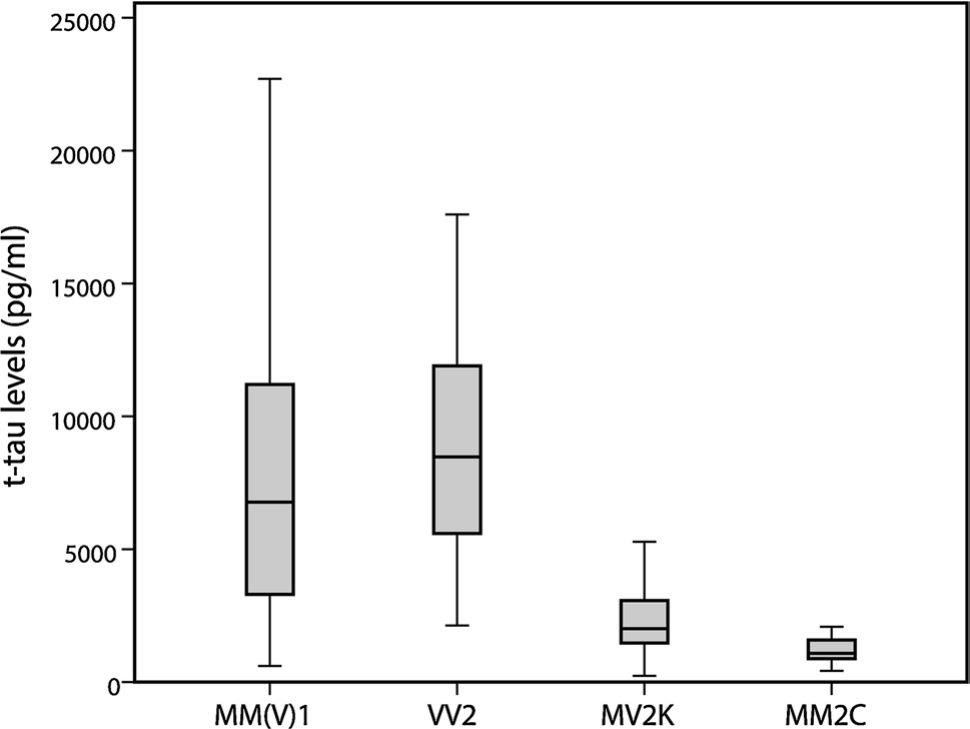
Effect of sCJD subtype on t-tau CSF levels. T-tau protein levels were significantly higher in sCJD VV2 (*n* = 29), and MM(V)1 (*n* = 111) subtypes than in the MV2K (*n* = 26) and MM2C (*n* = 10) subtypes (MM(V)1 vs. MV2K *p* < 0.001; MM(V)1 vs. MM2C *p* < 0.001; VV2 vs. MV2K *p* < 0.001; VV2 vs. MM2C *p* < 0.001)

Comparison of t-tau levels among the different sCJD groups largely correlated with the diagnostic sensitivity rates outlined above. Specifically, VV and MM homozygotes showed significantly higher tau levels than MV patients (MM vs. MV *p* < 0.001; VV vs. MV *p* < 0.001). Furthermore, t-tau protein levels were significantly higher in sCJD subtypes VV2 (8478, IQR 5422–12350) and MM(V)1 (6848, IQR 3302–11300) than in the MV2K (2012, IQR 1405–3083), MM2C (1084, IQR 858–1713) and MM2T subtypes (310, 630, and 1377 pg/ml in the three cases examined) (Fig. 1) (MM(V)1 vs. MV2K *p* < 0.001; MM(V)1 vs. MM2C *p* < 0.001; VV2 vs. MV2K *p* < 0.001; VV2 vs. MM2C *p* < 0.001). Finally, patients carrying the V210I-129M haplotype showed significantly increased CSF t-tau levels (6735, IQR 3412–11000) compared to subjects carrying the E200K-129M haplotype (2205, IQR 1344–3114) (*p* < 0.001).

Among the false-positive cases, the diagnostic category CNS malignancy had the highest relative incidence, followed by encephalitis, vascular dementia and AD (Table 3; Suppl. Table 3).

### RT-QuIC assay

The results of the RT-QuIC assay for the different diagnostic groups are summarised in Table 1. At the chosen threshold 82.7% of definite sCJD, 91.3% of definite gCJD, 79.4% of probable sCJD and 75.9% of possible sCJD cases had a positive RT-QuIC assay (Table 1). The overall calculated test sensitivity (all CJD vs. non-CJD) was 82.1% and the test specificity was 99.4%, while the test PPV and NPV were, respectively, 99.0 and 84.4%.

**Fig. 2:**
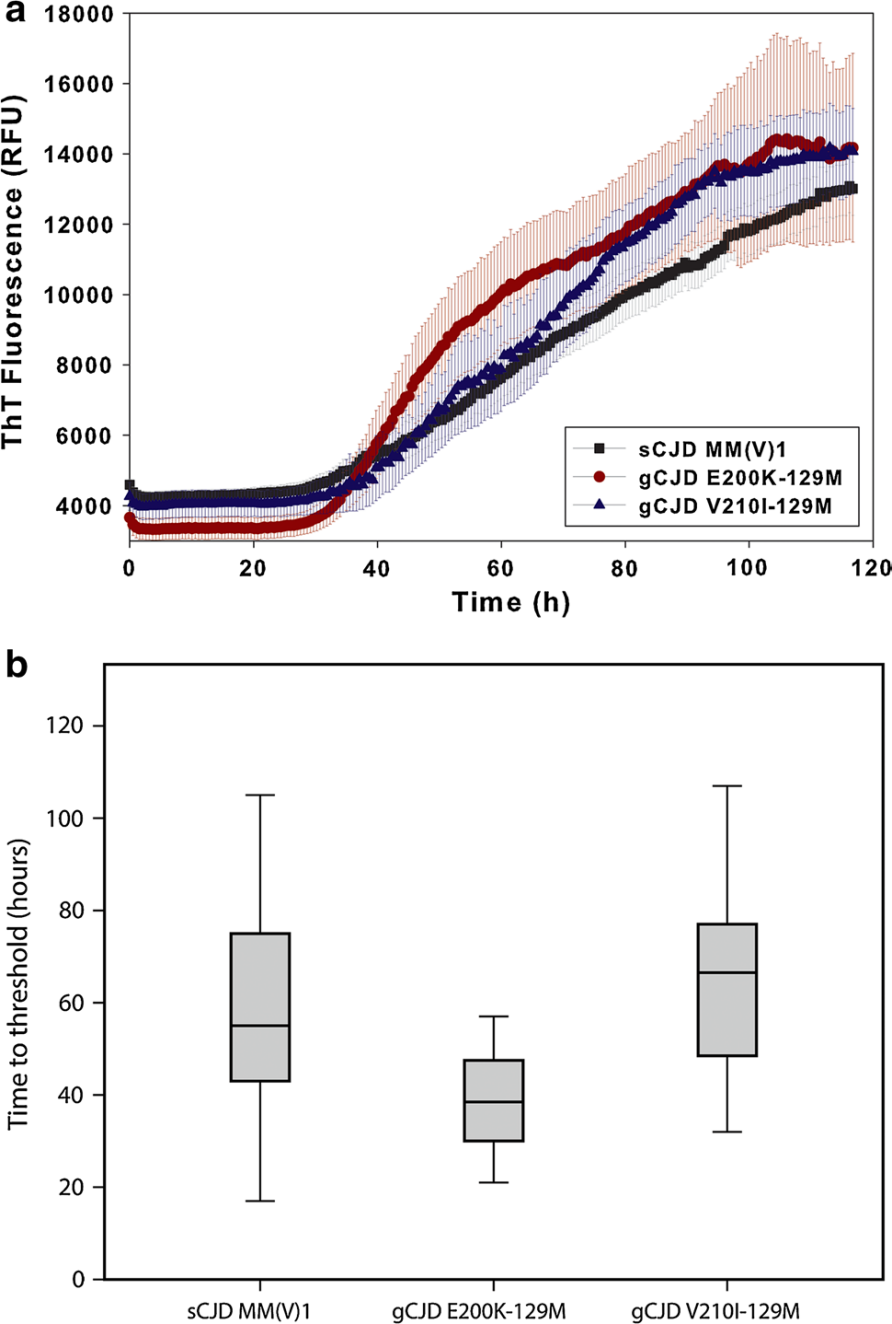
Time course of prion seeding detected by RT-QuIC in CSF of sCJD MM(V)1, gCJD E200K-129M and gCJD V210I-129M. **a** Averaged RT-QuIC kinetics for sCJD MM1 and gCJD (E200K-129M or V210I-129M haplotypes) CSF samples. Traces represent the mean (±standard error of the mean [SEM]) of ThT fluorescence for CSF specimens from representative groups of patients with sCJD MM1 (*black*, *n* = 50), gCJD E200K-129M (*red*, *n* = 10) and gCJD V210I-129 M (*blue*, *n* = 10). **b** Box plots of the times to threshold (as defined in the “Materials and methods”) for sCJD MM(V)1 (*n* = 90), gCJD E200K-129M (*n* = 16) and gCJD V210I-129M (*n* = 20) CSFs with a positive RT-QuIC assay. Statistically significant differences were found when comparing gCJD E200K-129M vs. sCJD MM(V)1 (*p* < 0.001) or vs. gCJD V210I-129M (*p* < 0.001)

RT-QuIC sensitivity in sCJD varied according to the codon 129 genotype and was higher in MM (84.2%) than in MV (72.2%) or VV (79.5%) subjects. Consistently, subjects affected by the prevalent MM(V)1 subtype showed a higher sensitivity than those affected by any of the atypical disease subtypes (Table 2). However, there was no relationship between codon 129 genotype or disease subtype and either the lag phase or the maximal fluorescence signal response recorded during the RT-QuIC analysis among positive cases (data not shown). Furthermore, patients carrying the E200K-129M haplotype had a higher RT-QuIC sensitivity (100%) than those carrying V210I-129M (95.2%), which correlated with a significantly shorter lag phase in the former group (Fig. 2). Among the subjects carrying other rarer mutations, only the patient with the V203I substitution gave a positive response (Table 1). Genetic CJD patients carrying E200K-129M showed a slightly increased maximal ThT signal (14289, IQR 9742–16006) compared to both gCJD carrying V210I-129M (12720, IQR 9095–16696) and sCJD MM(V)1 subjects (12140, IQR 9400–15787), but the difference did not reach statistical significance.

Overall, the specificity of RT-QuIC was substantially better than that of CSF 14-3-3 or t-tau. Only two CSF samples from the non-CJD group gave a positive RT-QuIC response. This first CSF was from a patient with a RPD and a clinical diagnosis of paraneoplastic syndrome (a metastatic breast carcinoma was found) who had a positive 14-3-3, increased t-tau levels (1588 pg/ml) and a total disease duration of 4 months. The second patient, who was lost to follow-up, was referred with a clinical diagnosis of suspected FTD (clinical symptoms, neuropsychological tests and FDG-PET demonstrated a predominant fronto-temporal involvement) after a relatively rapid clinical course of about 1.5 years. Results of diagnostic investigation included a negative standard (no DWI sequence) cerebral MRI, a negative 14-3-3 test, increased t-tau (1362 pg/ml) and p-tau levels (154 pg/ml) and reduced Aβ42 (369 pg/ml).

### CSF p-tau and the t-tau/p-tau ratio

CSF p-tau levels were significantly higher in AD patients (87, IQR 72–117; *n* = 85) than in CJD (49, IQR 36–67; *n* = 294) and the other non-CJD patients (48.5, IQR 30.8–75; *n* = 226) (AD vs. CJD *p* < 0.001; other non-CJD vs. CJD *p* = 0.744; AD vs. other non-CJD *p* < 0.001).

**Fig. 3:**
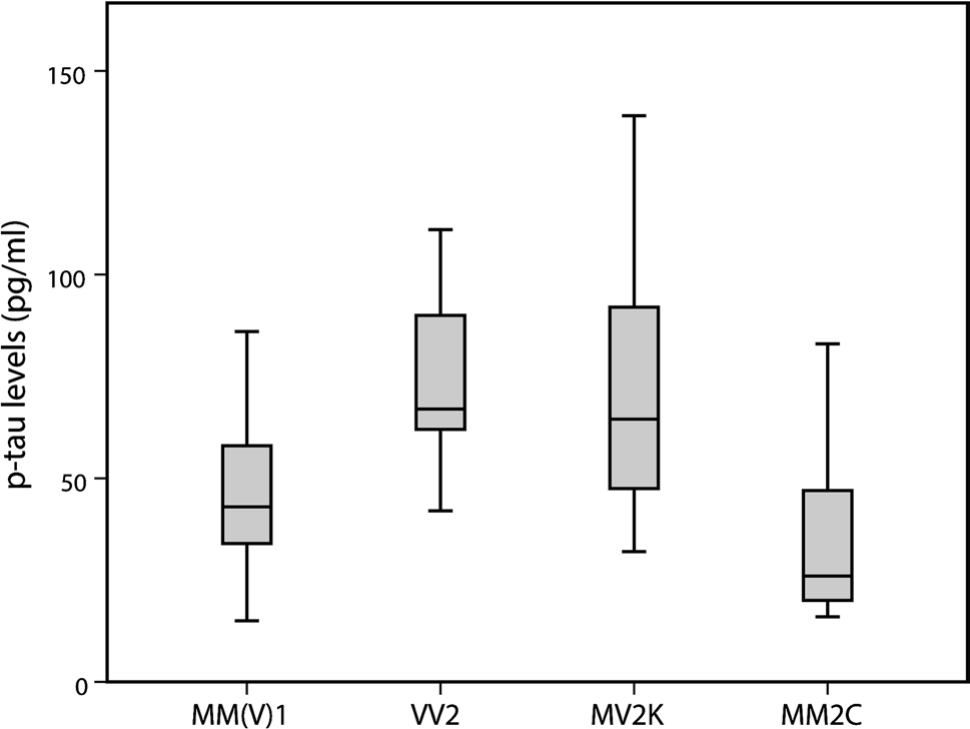
Effect of sCJD subtype on p-tau CSF levels. VV2 (*n* = 27) and MV2K (*n* = 24) patients showed significantly higher p-tau levels than MM(V)1 (*n* = 103) or MM2C (*n* = 10) cases (VV2 vs. MM(V)1 *p* < 0.001; MV2K vs. MM(V)1 *p* < 0.001; VV2 vs. MV2K *p* = 0.308; VV2 vs. MM2C *p* < 0.001; MV2K vs. MM2C p < 0.001)

Interestingly, p-tau levels varied significantly among sCJD subtypes, with both VV2 (67, IQR 62–94; *n* = 27) and MV2K (64.5, IQR 46.8–94; *n* = 24) patients showing significantly higher levels than MM(V)1 (43, IQR 34–58; *n* = 103) (*p* < 0.001) or MM2C cases (26, IQR 19–52; *n* = 10) (*p* < 0.001) (Fig. 3). In the gCJD group, there were no significant differences in p-tau levels between V210I-129M and E200K-129M carriers (*p* = 0.111), while the single case of VPSPr showed one of the highest CSF p-tau levels of the prion disease group (140 pg/ml).

**Fig. 4:**
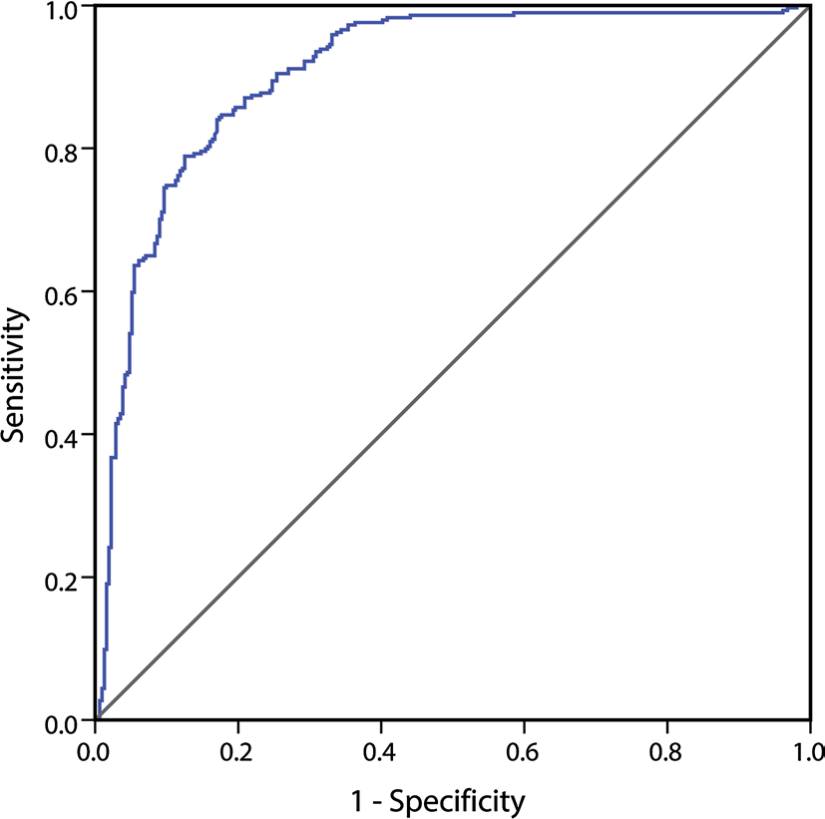
ROC analysis for t-tau/p-tau ratio. The t-tau/p-tau ratio achieved an area under the curve of 0.907 ± 0.013 in the discrimination of all CJD (*n* = 294) from all non-CJD (*n* = 311) with 84% sensitivity and 83% specificity, using a cut-off value of 31.8

As previously reported, CJD patients present with a significant increase in the t-tau/p-tau ratio (97.5, IQR 42.6–185.7; *n* = 294) in comparison to both AD (7.9, IQR 6.7–10.2; *n* = 85) and other non-CJD (15.2, IQR 8.0–31.2; *n* = 226) subjects (CJD vs. AD *p* < 0.001; CJD vs. non-CJD *p* < 0.001; AD vs. non-CJD *p* < 0.001). Results from the ROC analysis for t-tau/p-tau ratio are illustrated in Fig. 4. The t-tau/p-tau ratio achieved an AUC of 0.907 ± 0.013 in the discrimination of CJD from all non-CJD with 84% sensitivity and 83% specificity, using a cut-off value of 31.8. The ROC analysis for t-tau alone on the same patient population (*n* = 294 CJD and 311 non-CJD) showed a similar AUC value (0.904 ± 0.013). However, the calculated AUC of the t-tau/p-tau ratio in the distinction between CJD and non-CJD, after excluding the AD patients, was lower than the one for t-tau alone in the same group of patients (0.876 ± 0.016 vs. 0.892 ± 0.015). Details on the numbers of non-CJD samples used for the p-tau assay for each diagnostic category are provided in Suppl. Table 4.

We also found statistically significant differences in the t-tau/p-tau ratio among the different sCJD subtypes. The MM(V)1 group showed the highest values (158.41, IQR 74.0–220.9; *n* = 103), followed by the VV2 (110.8, IQR 90.7–148.8; *n* = 27), MM2C (49.6, IQR 19.2–59.1; *n* = 10), and MV2K (26.4, IQR 20.9–51.7; *n* = 24) groups (MM(V)1 vs. MV2K *p* < 0.001; MM(V)1 vs. MM2C *p* < 0.001; VV2 vs. MV2K *p* < 0.001).

### Correlation between CSF p-tau levels and tau neuropathology

#### Analysis of prion-related tau pathology

**Fig. 5:**
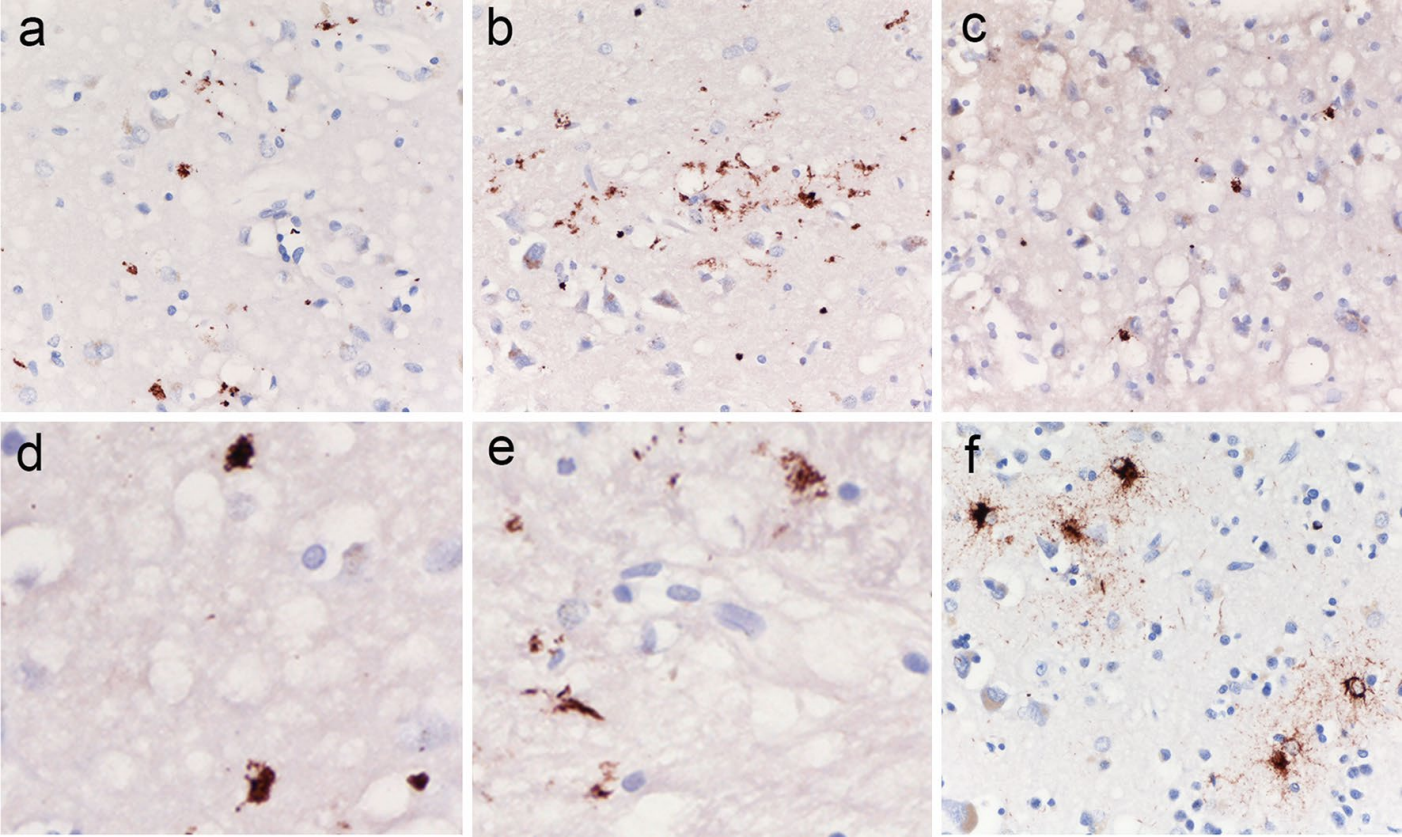
Immunohistochemistry for p-tau (AT8 antibody) in brain regions showing spongiform change in sporadic CJD VV2, MV2K, and VPSPr. Dot- and stub-like tiny tau deposits in the deep layers of the temporal neocortex (**a** = ×400; **d** = ×1000), and in the thalamus (**c** = ×400; **e** = ×1000) of a sCJD VV2 case, and in the anterior striatum (**b** = ×400) of a sCJD MV2K; cluster of granular-fuzzy astrocytes in the cerebral cortex of the VPSPr case (**f** ×400)

**Fig. 6:**
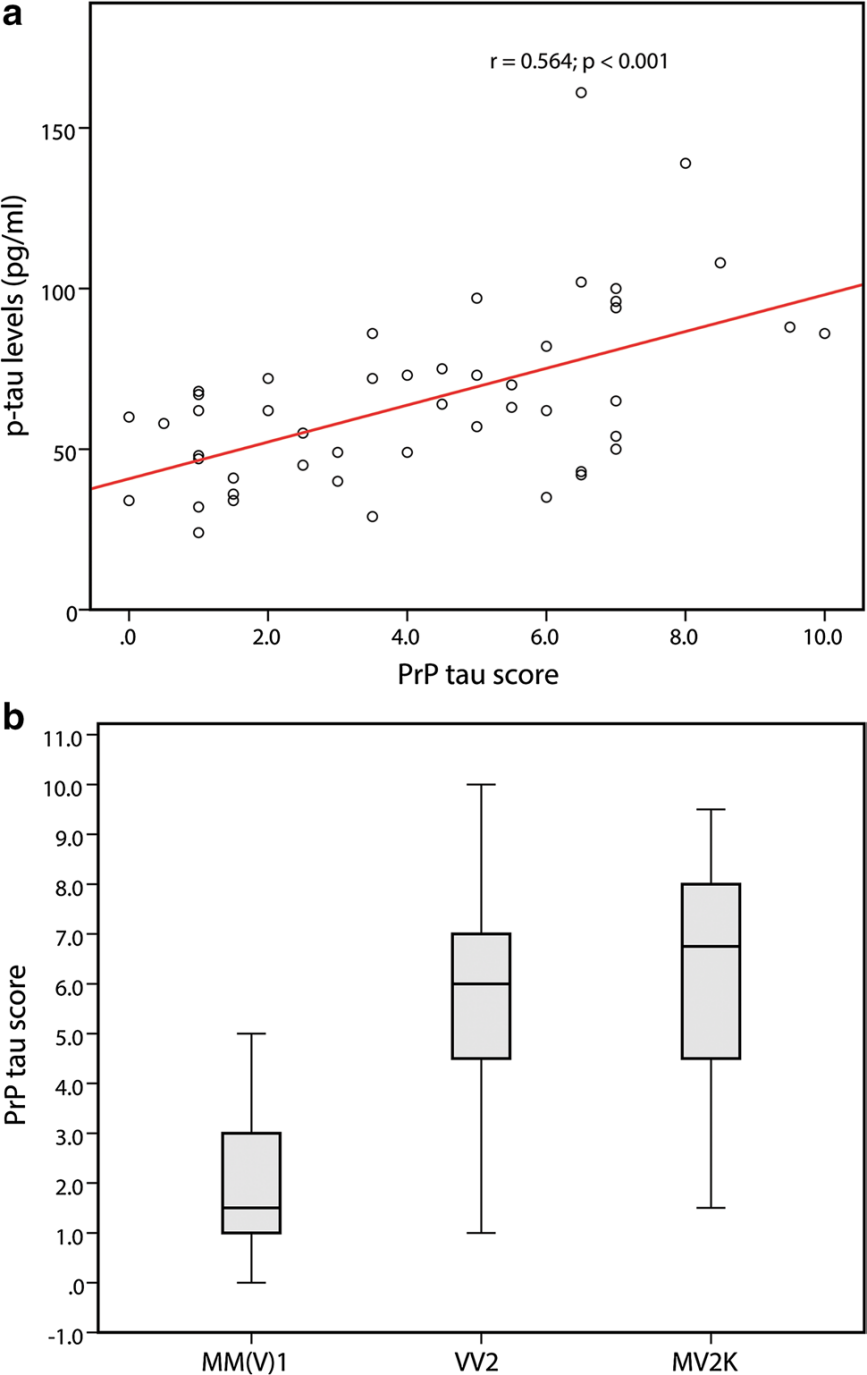
Correlation between CSF p-tau levels and PrP-tau score. **a** A significant positive correlation is seen between p-tau levels in the CSF and the amount of prion-specific tau deposition in the brain (*r* = 0.564, *p* < 0.001; *n* = 50); **b** Brains from both VV2 (6, IQR 4–7; *n* = 21) and MV2K (7, IQR 4–8; *n* = 10) cases showed a significantly higher score of positive immunoreactivity than those from MM(V)1 cases (1.5, IQR 1–3; *n* = 19) (MM(V)1 vs. VV2 *p* < 0.001; MM(V)1 vs. MV2K *p* < 0.001; MV2K vs. VV2 *p* = 0.699)

To search for abnormal tau deposits specifically related to CJD pathology, we evaluated p-tau immunoreactivity in brain regions showing the most prominent spongiform change in subject with no or only minimal AD-related changes. An initial screening revealed the presence of a dot- or stub-like positive immunoreactivity (Fig. 5) in most of the examined cases. However, the number and, to some extent, the size of the abnormal tau-deposits varied significantly among cases, often irrespectively of the degree of spongiform change, and was clearly correlated with the CJD type (see below). Furthermore, in all CJD types, the dot- or stub-like p-tau deposits were rarely detected, or not seen at all, in the cases with severe neuronal/neurophil loss and reactive gliosis. In a selected group of 50 cases with typical spongiform change and mild to moderate neuronal loss, we found a highly significant positive correlation between the amount of this prion-specific tau deposition and p-tau levels in the CSF (*r* = 0.564, *p* < 0.001; *n* = 50) (Fig. 6a). Consistently, brains from both VV2 (6, IQR 4–7; *n* = 21) and MV2K (7, IQR 4–8; *n* = 10) cases showed a significantly higher score of positive immunoreactivity than those from MM(V)1 cases (1.5, IQR 1–3; *n* = 19) (MM(V)1 vs. VV2 *p* < 0.001; MM(V)1 vs. MV2K *p* < 0.001; MV2K vs. VV2 *p* = 0.699) (Fig. 6b). The correlation between the distribution of spongiform change and the presence of the abnormal p-tau deposits was particularly striking in the cerebral cortex of VV2 cases in which both lesions often co-distributed in a laminar pattern only involving the deep cortical layers (Fig. 5a and d). The analysis of 5 genetic CJD cases (E200K and V210I mutations) did not reveal significant differences in the extent of prion-specific tau deposition in comparison to the sporadic cases of the same MM(V)1 type.

#### Analysis of AD-related tau pathology

**Fig. 7:**
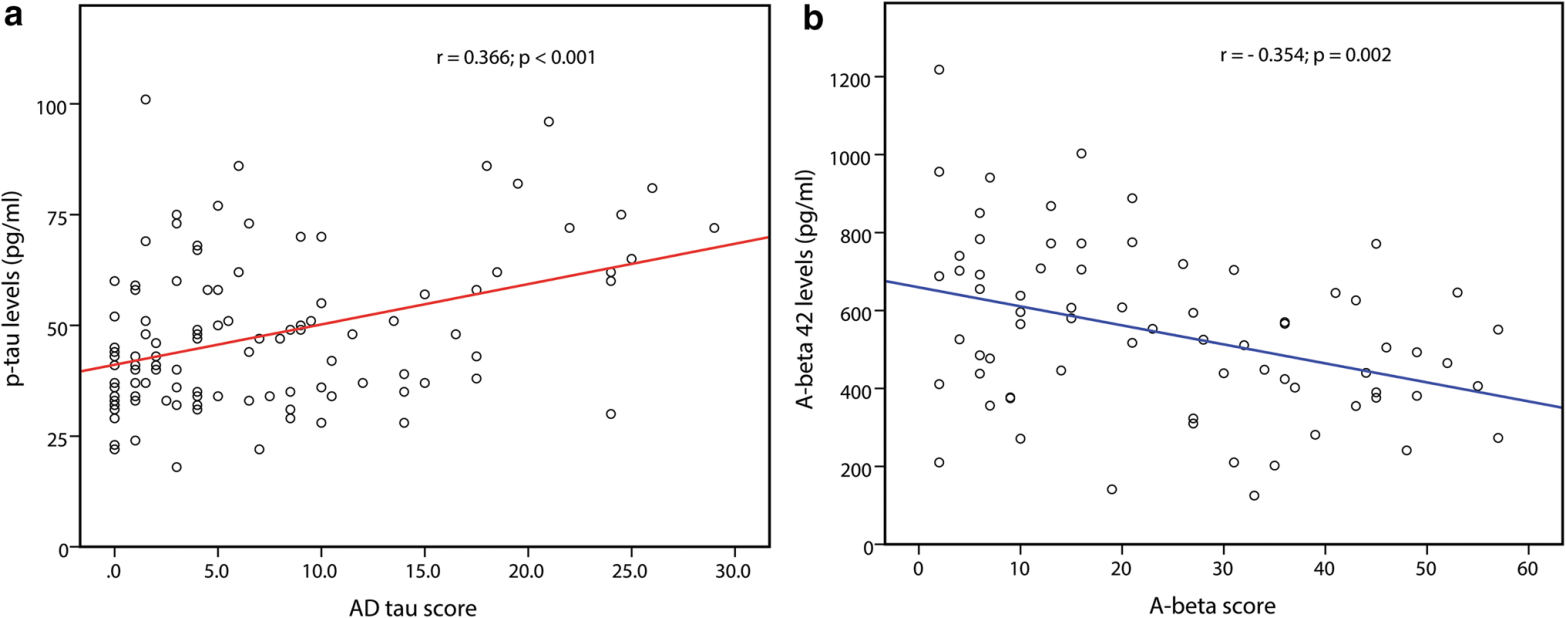
Correlations between CSF p-tau and Aβ42 levels and AD-related neuropathology in CJD MM(V)1. **a** A significant positive correlation is seen between CSF p-tau levels and the AD-pathology score (*r* = 0.366, *p* < 0.001; *n* = 109). **b** A significant inverse correlation is seen between CSF Aβ42 levels and the Aβ pathology score (*r* = −0.354, *p* = 0.002; *n* = 73)

To determine the effect of AD-related tau pathology to the CSF levels of p-tau we focused on the CJD MM(V)1 cases, the largest homogeneous group available (*n* = 109 brains) and the one showing the least amount of prion-specific tau pathology (see above). The median time interval between lumbar puncture and death in this group was 1.3 months (IQR 0.5–2.5). Within the group, we found a significant positive correlation between CSF p-tau levels and the AD-pathology score (*r* = 0.366, *p* < 0.001) (Fig. 7a). Thus, CSF p-tau levels in CJD also reflect the extent of co-morbid neurofibrillary AD pathology. We also correlated CSF p-tau concentrations with Braak stages and found that stages III–V were linked to abnormal, above threshold (60 pg/ml), p-tau CSF levels in the majority (71%) of cases (69, IQR 54–81; *n* = 14), whereas the majority (84%) of brains with Braak stage I–II were associated with CSF values of p-tau within the range of controls (48, IQR 35–57; *n* = 37), although with slightly higher median values than those in Braak stage 0/+ (40, IQR 33–51; *n* = 58). Consistently with the CSF findings, no significant differences (*p* = 0.529) in the AD-pathology score were seen between sporadic (4, IQR 1–10.5; *n* = 95) and genetic (5, IQR 0–6.5; *n* = 14) CJD cases of the MM(V)1 type.

To address whether a higher degree of neuronal AD related tau pathology (in comparison to the other subtypes), besides the tiny neuritic tau profiles, could have contributed to the increased of p-tau levels in sCJD VV2 and MV2K, we also compared the relative distribution of Braak stages and of the mean AD-related score among the major disease subtypes. The finding of a trend towards a higher degree of AD pathology in the MM(V)1 (see Suppl. Table 5) excluded this possibility. To the same aim, we also screened the posterior hippocampus for glial tau pathology and found evidence for ARTAG (subependymal type in focal clusters) in a limited number of CJD cases (7 out of 158, 4.5%), belonging to both the MM(V)1 and VV2 subtypes (see Suppl. Table 5). Thus, the relative rarity of ARTAG in both our series, and the one studied by Kovacs et al. (6.6% in a CJD population with a mean age of 72 yrs) [36], with no apparent difference in frequency between MM(V)1 and VV2/MV2K cases, also excludes a significant specific contribution of ARTAG to the increased CSF p-tau levels we have found in VV2 and MV2K cases.

### CSF Aβ42 and correlation with the Aβ load in the brain

Overall, the Aβ42 concentration was significantly lower in AD patients (326, IQR 274–394; *n* = 61) than in the CJD group (508, IQR 360–717; *n* = 164) and the other non-CJD groups (514, IQR 279–799; *n* = 60) (AD vs. CJD *p* < 0.001; AD vs. non-CJD *p* < 0.001; other non-CJD vs. CJD *p* = 0.926). Among CJD samples there was no significant difference among sCJD subtypes or gCJD cases carrying the V210I-129M or the E200K-129M haplotypes.

**Fig. 8:**
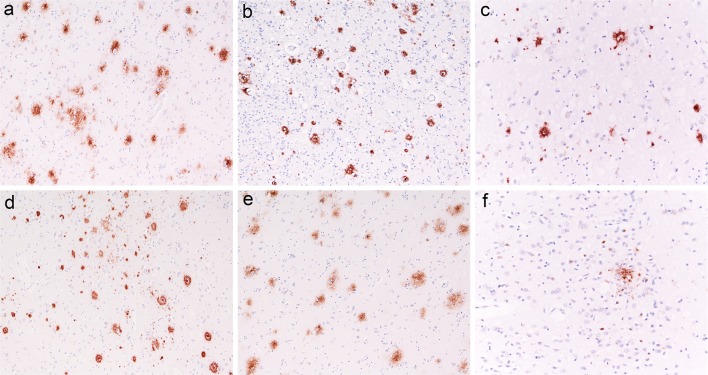
Examples of imperfect correlation between CSF Aβ42 levels and extent of Aβ brain deposits in CJD. Various Aβ aggregates (plaques) in CJD brains (**a**–**f**); **a** sCJD MM1+2C, temporal cortex, Thal phase III, AD-score = 31, CSF Aβ42 = 704 pg/ml, magnification ×100; **b**, **c** sCJD MV2K, parietal cortex (**b**) and striatum (**c**), Thal phase III, AD-score = 45, CSF Aβ42 = 771 pg/ml, magnification ×100 (**b**) or ×200 (**c**); **d**, **e** sCJD VV2, parietal cortex (**d**) and amygdala (**e**), Thal phase IV, AD-score = 53, CSF Aβ42 = 646 pg/ml, magnification ×100; sCJD MM1 (**f**), Thal phase I, AD-score = 2, CSF Aβ42 = 411 pg/ml, magnification ×200

Among the definite sporadic and genetic CJD cases examined for the correlation between CSF Aβ42 concentration and Aβ brain deposits (*n* = 118), the median time interval between lumbar puncture and death was 2 months (IQR 1–4.4). Forty-five brains showed no Aβ deposits (score = 0). In this cohort, the median Aβ42 level was 579 pg/ml (IQR 369–754), with 38% of patients (17/45) having CSF Aβ42 levels below the threshold of 450 pg/ml, whereas in those with Aβ brain deposits (*n* = 73), the Aβ score showed a significant inverse correlation with CSF Aβ42 levels (*r* = −0.354, *p* = 0.002) (Fig. 7b). Thus, CSF Aβ42 levels in CJD reflect both CJD-related pathology and the extent of co-morbid AD pathology. We also correlated CSF Aβ42 levels with the Thal phase, and found that only the brains with advanced phases (IV or V) showed abnormal, below threshold, Aβ42 CSF levels in almost all cases (82%; 402, IQR 277–479; *n* = 17), whereas the majority (68%) of those in phases I–III showed CSF values of Aβ42 within the range of controls (575, IQR 439–703; *n* = 54) (Fig. 8). Finally the two cases with pure (mild) CAA and no parenchymal Aβ deposits showed Aβ42 CSF levels within the normal range (956 and 550 pg/ml).

### Correlation of CSF and brain findings in VPSPr

The high CSF p-tau levels (140 pg/ml) and the low Aβ42 levels (402 pg/ml) in the single case of VPSPr examined in this study correlated with a significant brain Aβ load (Thal phase IV, Aβ score 37) and a mixed brain tauopathy comprising features of PART/AD (Braak stage III, AD-tau score 15), prion-related tauopathy (PrP-tau score 3.5) and ARTAG. Specifically, the following types of ARTAG were seen: gray matter type; regions involved: MTL, lobar, subcortical, and brainstem; extent: occasional, solitary or in focal clusters (Fig. 5f); plus laminar subpial type; regions involved: MTL, lobar, subcortical, and brainstem; extent: numerous, multifocal; plus subependymal type, regions involved: MTL; extent: in focal clusters; plus perivascular and white matter types; regions involved: MTL, lobar, subcortical, and brainstem; extent: in focal clusters of various size.

### Effect of age, sex, timing of lumbar puncture, and disease duration on CSF biomarkers

Demographic characteristics for definite, probable and possible CJD are provided in Suppl. Table 6. In the group of definite and probable CJD patients, there was no effect of sex on Aβ42 (*p* = 0.123), p-tau levels (*p* = 0.496) and RT-QuIC results (*p* = 0.533). In the same group, when stratifying p-tau, Aβ42, RT-QuIC results by age, there was no effect of age on Aβ42 (*p* = 0.061), p-tau (*p* = 0.115) levels and RT-QuIC results (*p* = 0.384) (see Suppl. Table 7). In the group of definite sporadic MM(V)1 there was no effect of time from clinical onset to LP and of time from LP to death on Aβ42 (*p* = 0.691; 0.256), t-tau (*p* = 0.815; 0.225), p-tau levels (*p* = 0.707; 0.248), 14-3-3 (*p* = 0.238; 0.346), and RT-QuIC results (*p* = 0.210; 0.673) (see Suppl. Table 8). In the same group, there was no effect of disease duration on Aβ42 (*p* = 0.566), t-tau (*p* = 0.636), p-tau levels (p = 0.515), 14-3-3 (*p* = 0.430) and RT-QuIC results (*p* = 0.930) (see Suppl. Table 8).

## Discussion

The present study reports the results of a comprehensive analysis of all major available CSF biomarkers for the differential diagnosis of CJD in a large unselected clinical population reflecting clinical practice, taking also into account the effect of the disease subtype on diagnostic accuracy and the influence of tau and Aβ brain pathology on p-tau and Aβ42 CSF levels.

Our results confirm the very high specificity of RT-QuIC for the diagnosis of sCJD in a large series of CJD and non-CJD CSF samples. However, they also provide, for the first time, convincing evidence for a lower sensitivity of the assay in atypical subtypes of the disease characterised by PrP^Sc^ type 2 (e.g.,VV2, MV2K, and MM2C). The negative results we repeatedly obtained with the CSF samples from a patient affected by VPSPr and one from a GSS patient carrying the D202N mutation [P. Parchi, personal communication] are also consistent with this finding. Thus, as with CSF protein surrogate markers, the in vitro conversion reaction exploited by RT-QuiC is significantly affected by the neurobiological heterogeneity of human prions.

Recent studies using a different recombinant PrP substrate or olfactory mucosa brushings instead of CSF as an alternative seed reported a 96–97% sensitivity of the RT-QuIC assay for the diagnosis of sCJD [45, 46]. However, although very promising, these novel modified RT-QuIC assays have, to date, only been applied to sCJD cases largely represented by typical CJD patients affected by the MM(V)1 type. Furthermore, at variance with the methodology used in the present study [42], the modified assays have not been, as yet, validated in the inter-laboratory setting.

The reasons behind the variability in seeding activity among CSF samples from CJD patients remain, unfortunately, partially unclear. Indeed, besides the above-mentioned correlation with the disease subtype, we failed to reveal other common features among the CSF samples giving a negative RT-QuIC result. In particular, there was no significant correlation with age at onset, disease duration at the time of lumbar puncture, or with the results of both conventional protein assays and brain MRI. Future studies should further address this issue and establish, in the first instance, whether seeding activity correlates or not with PrP^Sc^ levels. Nevertheless, our observation of a higher conversion activity of the samples from gCJD patients carrying the E200K-129M haplotype (and PrP^Sc^ type 1), in comparison to those of the sCJD MM(V)1 group, which is confirmatory of the results of a previous study [19], supports the view of a direct role of PrP^Sc^ in seeding rec-PrP conversion and aggregation.

Regarding the more conventional CSF surrogate diagnostic markers, such as proteins t-tau and 14-3-3, our data demonstrate that t-tau is (moderately) superior to 14-3-3 in the diagnosis of sCJD. More specifically, t-tau yields a lower number of false positive results, especially in cases suffering from inflammatory-related conditions and subacute dementias; moreover, it has a higher sensitivity than 14-3-3 for the sCJD MV2K type. These results, which are in line with those reported by Hamlin et al. [30], together with the notion that the t-tau assay has technical advantages with respect to the standard western blot 14-3-3 assay, suggest a change in the current recommendations to prioritise t-tau analysis over 14-3-3. Interestingly, t-tau appears to be less accurate than 14-3-3 only in the differential diagnosis with AD; however, in cases where the differential diagnosis with AD is an issue, biomarker analysis can also take advantage of Aβ42 and p-tau and possibly total-PrP dosage, allowing for the calculation of t-tau/p-tau and other ratios based on different combinations of these four biomarkers, which have been reported to significantly improve diagnostic accuracy in such cases [1, 21, 67]. Outside this specific clinical scenario (CJD vs rapidly progressive AD), however, our data do not support the additional diagnostic value of t-tau/p-tau ratio with respect to t-tau alone. This lower diagnostic performance of t-tau/p-tau ratio compared to what is generally reported in the literature [9, 12, 21, 60, 67] likely reflects differences in the characteristics of the examined patient populations. Indeed, while previous studies mainly analysed the diagnostic accuracy of the t-tau/p-tau ratio in the differential diagnosis between typical CJD and AD or between CJD and large clinically unselected populations of patients with dementia, our study focused on patients referred for the suspicion of CJD and included a relatively high number of CJD cases of less common subtypes such as VV2, MV2K and MM2C, showing a significantly lower t-tau/p-tau ratio than the typical CJD MM(V)1.

Our results further underline the profound implications that the phenotypic heterogeneity of the disease has on diagnostic criteria and the overall clinical approach to a patient suspected of having CJD. Indeed, as also clearly revealed by our study, the sensitivity of both surrogate protein markers and RT-QuIC varies significantly according to the CJD type. Given that knowledge about the patient’s *PRNP* codon 129 genotype, one of the two major molecular determinants of the disease type, can be obtained from a blood sample, it is crucial to include codon 129 genotyping in the diagnostic work-up of patients with suspected CJD. This information, combined with those obtained from the patient’s clinical history and neurological examination, which is usually able to discriminate between a typical (MM(V)1) or atypical (all the others) disease subtype are crucial for the correct interpretation of the results of CSF assays. The most striking example of the importance of codon 129 genotyping for the clinical diagnosis of CJD concerns the VV2 subtype, the second most common sCJD type [53]. Given the relatively uniform and consistent clinical phenotype early in the disease course in these subjects, and the 100% sensitivity of standard CSF assays, the finding of VV at codon 129 in such a clinical scenario is highly confirmatory of the diagnosis of sCJD, even in cases with a negative RT-QuIC result. In contrast, the finding of a negative 14-3-3 test and/or t-tau levels below 1250 pg/ml in a codon 129 VV patient will exclude the diagnosis of sCJD VV2 with a 100% NPV.

One of the major issues in the field of diagnostic biomarkers concerns their correlation with the underlying brain pathology. Besides the analyses of the effect of CJD subtypes on all biomarkers analysed, in this study we have correlated neuropathological and CSF findings for both p-tau and Aβ. Our findings of increased CSF p-tau concentrations in sCJD types VV2 and MV2K, (both caused by CJD strain V2), is of particular significance in this respect. Indeed, although the CSF tau profile in CJD patients usually combines excessively high t-tau with relatively low p-tau concentrations [21, 57, 67, 68], we and other authors have clearly shown that CSF p-tau levels can be significantly raised in prion disease too, owing, once again, to the heterogeneity of the disease. In previous studies, variant CJD subjects showed raised CSF p-tau in most cases [29], and elevated levels of CSF p-tau have also been detected in VPSPr and GSS [56], as also shown in individual cases in the present study. Interestingly, a secondary tauopathy characterised by brain deposition of abnormal p-tau aggregates has been demonstrated in all these atypical phenotypes of prion disease by post-mortem studies [26, 27, 31, 58], although the data on VPSPr are very limited. Thus, our CSF findings raise the question of whether a tauopathy in response to PrP^Sc^ deposition also develops in classic CJD, especially in the VV2 or MV2K subtypes. Initial studies were either negative [27 or led to the conclusion that only prion diseases associated with PrP-amyloid plaques develop p-tau deposits, whereas those characterised by synaptic PrP deposits do not [66]. More recently, Reiniger et al. [59] demonstrated that a tiny punctate (also described as rod-, dot-, or stub-shaped), PrP-specific, p-tau immunoreactivity, positively correlating with PrP^Sc^ burden in the neocortex, is consistently found in sCJD brains, irrespective of the molecular subtypes and the AD-related associated pathology. Consistently, in a recent study by Kovacs et al. [36] these small neuritic profiles were regarded as the most frequent type of tau immunoreactivity in CJD brains. Pursuing the findings of these studies we demonstrated here, for the first time, that in classic CJD the prion-specific secondary tauopathy especially affects the sCJD subtypes linked to the V2 strain (e.g., VV2 and MV2K) and, most significantly, that in such cases the tauopathy is severe enough to determine a significant increase in p-tau levels in the CSF. Furthermore, we reported the detection of prion specific p-tau dots in VPSPr, a finding we have confirmed in a second case, for whom we had no CSF [P. Parchi personal communication]. Small neuritic p-tau positive profiles have been, to date, only documented in the frontal cortex of a single case of VPSPr [4]. Overall, our results expand on but also help, to some extent, to understand some of the previous findings. Indeed, the fact that both VV2 and MV2K are sCJD types characterised by plaque-like PrP deposits or even PrP-amyloidosis (MV2K) and are associated, on average, with a higher PrP^Sc^ burden than those of the MM(V)1 type [49] somehow fits with the data of both Sikorska et al. [66] and Reiniger et al. [59]. Moreover, our observation that the PrP-related tau deposits were rarely detected in the cases with severe neurodegeneration also explains the previously described lack of correlation between tau deposits and disease duration [59].

Aβ42 concentration in the CSF of sCJD patients has been measured in a number of studies, but with inconsistent results [21, 34, 47, 71, 73, 75, 77]. Early studies, based on a limited number of cases, found significantly decreased Aβ42 levels in CSF of CJD patients with mean values comparable to those of AD patients and with no apparent correlation with either the *APOE* genotype or the number of Aβ-positive plaques in the brain [34, 47, 71]. More recently, however, Varges et al. [73] demonstrated a dose-dependent effect of *APOE*-*4* on the decrease in Aβ42 in the CSF of sCJD patients. Furthermore, other preliminary data suggest that Aβ42 levels in the CSF are significantly higher in sCJD than in AD. In the present study we definitely show, in a large group of sCJD cases, that (1) the CSF levels of Aβ42 are significantly higher in sCJD than in AD and that (2) about 60% of sCJD cases have a concentration of Aβ42 in the CSF within the range of controls. Nevertheless, our analyses of Aβ load in neuropathologically verified patients clearly shows that the decrease in CSF Aβ42 in CJD patients, although influenced by the co-morbid AD pathology, may occur irrespective of the plaque load. Thus, the more likely explanation for the reduced Aβ42 levels in a subgroup of sCJD patients seems to be a combined effect of age-related co-morbidity, influenced by the *APOE* genotype, and the effect of neuronal loss which can be very significant in CJD even a few months after clinical onset. An alternative explanation, which has been put forward in previous studies [47, 75], indicates that in pathologic conditions such as CJD a fraction of Aβ42 cannot be detected by conventional ELISA methods as epitopes may be masked.

Our findings have also implications for the issue of CSF biomarkers reliability in AD. Indeed, at variance with most neurodegenerative disorders including AD, brains affected by CJD and other rapidly progressive dementias provide the unique opportunity to correlate the CSF findings with the post-mortem neuropathology within a short time interval. Although our data confirm an overall positive correlation between p-tau levels in the CSF and the extent of brain tauopathy and an inverse correlation between Aβ42 levels and β-amyloid load, it must be emphasized that the correlation for Aβ42 and β-amyloid is rather gross. Of most significance in this respect, also given the “negative” effect of CJD on Aβ42 concentration in the CSF, is our finding of Aβ42 levels within the control range in 68% of CJD brains with a co-occurring significant Aβ deposition in the brain (Thal phases I–III). If confirmed in brains with other pathologies, this finding would suggest that the Aβ42 level in the CSF is far from being an optimal marker of the early phases of Aβ deposition in the brain in pre-clinical AD. Given that our data are limited to Aβ42, it will be important to expand the correlative analysis to other Aβ peptides such as Aβ40. Indeed, evidence suggests a better diagnostic performance of the Aβ42/40 CSF concentration ratio compared to the Aβ42 concentration alone [39, 76]. Furthermore, although AD typically affects both cerebral hemispheres to a similar degree, the fact we have only examined one hemisphere might be considered as a potential limitation of our study.

At variance with Aβ42, according to our data, CSF p-tau level represents a more reliable marker of brain AD-related tau pathology since it fails to consistently discriminate between patients with or without neurofibrillary pathology only when p-tau deposition is relatively mild and limited to the transentorhinal/entorhinal cortices (Braak stages I–II).

In summary, our study provides confirmatory and novel evidence for a significantly improved value of CSF biomarkers for the clinical diagnosis of CJD. Although RT-QuIC is the most promising assay, given its high specificity, t-tau remains the most important surrogate marker. Our data support a change in the current diagnostic criteria for CJD, indicating both RT-QuIC and t-tau as primary laboratory investigations to be implemented in suspected cases of CJD, in combination, when possible, with codon 129 genotyping. In cases of a negative RT-QuIC result and elevated t-tau in a patient with rapidly progressive cognitive impairment, the differential diagnosis between CJD and atypical AD is also supported by the combined analyses of CSF p-tau, Aβ42 and possibly total-PrP [1, 21]. Finally, in cases with a negative RT-QuIC result the determination of CSF t-tau in combination with codon 129 genotyping remains of importance for the clinical diagnosis of sCJD VV2.

Despite the significant advances, the diagnostic value of both CSF biomarkers and cerebral MRI remain very low in some rare atypical variants of sporadic prion disease, such as sporadic fatal insomnia (MM2T) and VPSPr. Efforts should continue in the attempt to recognise such atypical forms through disease surveillance and the neuropathological examination of suspected cases. Future studies should also aim to develop a type-specific RT-QuIC or other assay to discriminate the molecular subtypes of sCJD cases in vitam to bring the epidemiological surveillance of sCJD to the next level.

## Electronic supplementary material

Below is the link to the electronic supplementary material.

Supplementary material 1 (DOCX 42 kb)

## References

[1] Abu Rumeileh S, Lattanzio F, Stanzani Maserati M, Rizzi R, Capellari S, Parchi P (2017). Diagnostic accuracy of a combined analysis of cerebrospinal fluid t-PrP, t-tau, p-tau, and Aβ42 in the differential diagnosis of creutzfeldt-Jakob disease from Alzheimer’s disease with emphasis on atypical disease variants. J Alzheimers Dis.

[2] Alafuzoff I, Arzberger T, Al-Sarraj S, Bodi I, Bogdanovic N, Braak H, Bugiani O, Del-Tredici K, Ferrer I, Gelpi E, Giaccone G, Graeber MB, Ince P, Kamphorst W, King A, Korkolopoulou P, Kovács GG, Larionov S, Meyronet D, Monoranu C, Parchi P, Patsouris E, Roggendorf W, Seilhean D, Tagliavini F, Stadelmann C, Streichenberger N, Thal DR, Wharton SB, Kretzschmar H (2008). Staging of neurofibrillary pathology in Alzheimer’s disease: a study of the BrainNet Europe Consortium. Brain Pathol.

[3] Alafuzoff I, Thal DR, Arzberger T, Bogdanovic N, Al-Sarraj S, Bodi I, Boluda S, Bugiani O, Duyckaerts C, Gelpi E, Gentleman S, Giaccone G, Graeber M, Hortobagyi T, Höftberger R, Ince P, Ironside JW, Kavantzas N, King A, Korkolopoulou P, Kovács GG, Meyronet D, Monoranu C, Nilsson T, Parchi P, Patsouris E, Pikkarainen M, Revesz T, Rozemuller A, Seilhean D, Schulz-Schaeffer W, Streichenberger N, Wharton SB, Kretzschmar H (2009). Assessment of beta-amyloid deposits in human brain: a study of the BrainNet Europe Consortium. Acta Neuropathol.

[4] Assar H, Topakian R, Weis S, Rahimi J, Trenkler J, Höftberger R, Aboulenein-Djamshidian F, Ströbel T, Budka H, Yull H, Head MW, Ironside JW, Kovacs GG (2015). A case of variably protease-sensitive prionopathy treated with doxycyclin. J Neurol Neurosurg Psychiatry.

[5] Atarashi R, Satoh K, Sano K, Fuse T, Yamaguchi N, Ishibashi D, Matsubara T, Nakagaki T, Yamanaka H, Shirabe S, Yamada M, Mizusawa H, Kitamoto T, Klug G, McGlade A, Collins SJ, Nishida N (2011). Ultrasensitive human prion detection in cerebrospinal fluid by real-time quaking-induced conversion. Nat Med.

[6] Baldeiras IE, Ribeiro MH, Pacheco P, Machado A, Santana I, Cunha L, Oliveira CR (2009). Diagnostic value of CSF protein profile in a Portuguese population of sCJD patients. J Neurol.

[7] Beck J, Pittman A, Adamson G, Campbell T, Kenny J, Houlden H, Rohrer JD, de Silva R, Shoai M, Uphill J, Poulter M, Hardy J, Mummery CJ, Warren JD, Schott JM, Fox NC, Rossor MN, Collinge J, Mead S (2014). Validation of next-generation sequencing technologies in genetic diagnosis of dementia. Neurobiol Aging.

[8] Bertrand A, Brandel JP, Grignon Y, Sazdovitch V, Seilhean D, Faucheux B, Privat N, Brault JL, Vital A, Uro-Coste E, Pluot M, Chapon F, Maurage CA, Letournel F, Vespignani H, Place G, Degos CF, Peoc’h K, Haïk S, Hauw JJ (2009). Wernicke encephalopathy and Creutzfeldt-Jakob disease. J Neurol.

[9] Blennow K, Johansson A, Zetterberg H (2005). Diagnostic value of 14-3-3beta immunoblot and T-tau/P-tau ratio in clinically suspected Creutzfeldt-Jakob disease. Int J Mol Med.

[10] Braak H, Alafuzoff I, Arzberger T, Kretzschmar H, Del Tredici K (2006). Staging of Alzheimer disease-associated neurofibrillary pathology using paraffin sections and immunocytochemistry. Acta Neuropathol.

[11] Buerger K, Ewers M, Pirttilä T, Zinkowski R, Alafuzoff I, Teipel SJ, DeBernardis J, Kerkman D, McCulloch C, Soininen H, Hampel H (2006). CSF phosphorylated tau protein correlates with neocortical neurofibrillary pathology in Alzheimer’s disease. Brain.

[12] Buerger K, Otto M, Teipel SJ, Zinkowski R, Blennow K, DeBernardis J, Kerkman D, Schröder J, Schönknecht P, Cepek L, McCulloch C, Möller HJ, Wiltfang J, Kretzschmar H, Hampel H (2006). Dissociation between CSF total tau and tau protein phosphorylated at threonine 231 in Creutzfeldt-Jakob disease. Neurobiol Aging.

[13] Caobelli F, Cobelli M, Pizzocaro C, Pavia M, Magnaldi S, Guerra UP (2015). The role of neuroimaging in evaluating patients affected by Creutzfeldt-Jakob disease: a systematic review of the literature. J Neuroimaging.

[14] Capellari S, Strammiello R, Saverioni D, Kretzschmar H, Parchi P (2011). Genetic Creutzfeldt Jakob disease and fatal familial insomnia: insights into phenotypic variability and disease pathogenesis. Acta Neuropathol.

[15] Castellani RJ, Colucci M, Xie Z, Zou W, Li C, Parchi P, Capellari S, Pastore M, Rahbar MH, Chen SG, Gambetti P (2004). Sensitivity of 14-3-3 protein test varies in subtypes of sporadic Creutzfeldt-Jakob disease. Neurology.

[16] Chitravas N, Jung RS, Kofskey DM, Blevins JE, Gambetti P, Leigh RJ, Cohen ML (2011). Treatable neurological disorders misdiagnosed as Creutzfeldt-Jakob disease. Ann Neurol.

[17] Chohan G, Pennington C, Mackenzie JM, Andrews M, Everington D, Will RG, Knight RS, Green AJ (2010). The role of cerebrospinal fluid 14-3-3 and other proteins in the diagnosis of sporadic Creutzfeldt-Jakob disease in the UK: a 10-year review. J Neurol Neurosurg Psychiatry.

[18] Coulthart MB, Jansen GH, Olsen E, Godal DL, Connolly T, Choi BC, Wang Z, Cashman NR (2011). Diagnostic accuracy of cerebrospinal fluid protein markers for sporadic Creutzfeldt-Jakob disease in Canada: a 6-year prospective study. BMC Neurol.

[19] Cramm M, Schmitz M, Karch A, Zafar S, Varges D, Mitrova E, Schroeder B, Raeber A, Kuhn F, Zerr I (2015). Characteristic CSF prion seeding efficiency in humans with prion diseases. Mol Neurobiol.

[20] Crary JF, Trojanowski JQ, Schneider JA, Abisambra JF, Abner EL, Alafuzoff I, Arnold SE, Attems J, Beach TG, Bigio EH, Cairns NJ, Dickson DW, Gearing M, Grinberg LT, Hof PR, Hyman BT, Jellinger K, Jicha GA, Kovacs GG, Knopman DS, Kofler J, Kukull WA, Mackenzie IR, Masliah E, McKee A, Montine TJ, Murray ME, Neltner JH, Santa-Maria I, Seeley WW, Serrano-Pozo A, Shelanski ML, Stein T, Takao M, Thal DR, Toledo JB, Troncoso JC, Vonsattel JP, White CL, Wisniewski T, Woltjer RL, Yamada M, Nelson PT (2014). Primary age-related tauopathy (PART): a common pathology associated with human aging. Acta Neuropathol.

[21] Dorey A, Tholance Y, Vighetto A, Perret-Liaudet A, Lachman I, Krolak-Salmon P, Wagner U, Struyfs H, De Deyn PP, El-Moualij B, Zorzi W, Meyronet D, Streichenberger N, Engelborghs S, Kovacs GG, Quadrio I (2015). Association of cerebrospinal fluid prion protein levels and the distinction between Alzheimer disease and Creutzfeldt-Jakob disease. JAMA Neurol.

[22] Geschwind MD, Martindale J, Miller D, DeArmond SJ, Uyehara-Lock J, Gaskin D, Kramer JH, Barbaro NM, Miller BL (2003). Challenging the clinical utility of the 14-3-3 protein for the diagnosis of sporadic Creutzfeldt-Jakob disease. Arch Neurol.

[23] Geschwind MD, Shu H, Haman A, Sejvar JJ, Miller BL (2008). Rapidly progressive dementia. Ann Neurol.

[24] Geschwind MD, Tan KM, Lennon VA, Barajas Jr RF, Haman A, Klein CJ, Josephson SA, Pittock SJ (2008). Voltage-gated potassium channel autoimmunity mimicking creutzfeldt-jakob disease. Arch Neurol.

[25] Ghetti B, Tagliavini F, Kovacs GG, Piccardo P, Dickson D, Weller RO (2011). Gerstmann–Sträussler–Scheinker Disease. Neurodegeneration: the molecular pathology of dementia and movement disorders.

[26] Giaccone G, Tagliavini F, Verga L, Frangione B, Farlow MR, Bugiani O, Ghetti B (1990). Neurofibrillary tangles of the Indiana kindred of Gerstmann-Sträussler-Scheinker disease share antigenic determinants with those of Alzheimer disease. Brain Res.

[27] Giaccone G, Mangieri M, Capobianco R, Limido L, Hauw JJ, Haïk S, Fociani P, Bugiani O, Tagliavini F (2008). Tauopathy in human and experimental variant Creutzfeldt-Jakob disease. Neurobiol Aging.

[28] Gmitterová K, Heinemann U, Krasnianski A, Gawinecka J, Zerr I (2016). Cerebrospinal fluid markers in the differentiation of molecular subtypes of sporadic Creutzfeldt-Jakob disease. Eur J Neurol.

[29] Goodall CA, Head MW, Everington D, Ironside JW, Knight RS, Green AJ (2006). Raised CSF phospho-tau concentrations in variant Creutzfeldt-Jakob disease: diagnostic and pathological implications. J Neurol Neurosurg Psychiatry.

[30] Hamlin C, Puoti G, Berri S, Sting E, Harris C, Cohen M, Spear C, Bizzi A, Debanne SM, Rowland DY (2012). A comparison of tau and 14-3-3 protein in the diagnosis of Creutzfeldt-Jakob disease. Neurology.

[31] Head MW, Lowrie S, Chohan G, Knight R, Scoones DJ, Ironside JW (2010). Variably protease-sensitive prionopathy in a PRNP codon 129 heterozygous UK patient with co-existing tau, α synuclein and Aβ pathology. Acta Neuropathol.

[32] Jansen C, Parchi P, Capellari S, Ibrahim-Verbaas CA, Schuur M, Strammiello R, Corrado P, Bishop MT, van Gool WA, Verbeek MM, Baas F, van Saane W, Spliet WG, Jansen GH, van Duijn CM, Rozemuller AJ (2012). Human prion diseases in the Netherlands (1998-2009): clinical, genetic and molecular aspects. PLoS One.

[33] Josephs KA, Ahlskog JE, Parisi JE, Boeve BF, Crum BA, Giannini C, Petersen RC (2009). Rapidly progressive neurodegenerative dementias. Arch Neurol.

[34] Kapaki E, Kilidireas K, Paraskevas GP, Michalopoulou M, Patsouris E (2001). Highly increased CSF tau protein and decreased beta-amyloid (1–42) in sporadic CJD: a discrimination from Alzheimer’s disease?. J Neurol Neurosurg Psychiatry.

[35] Karch A, Hermann P, Ponto C, Schmitz M, Arora A, Zafar S, Llorens F, Müller-Heine A, Zerr I (2015). Cerebrospinal fluid tau levels are a marker for molecular subtype in sporadic Creutzfeldt-Jakob disease. Neurobiol Aging.

[36] Kovacs GG, Rahimi J, Ströbel T, Lutz MI, Regelsberger G, Streichenberger N, Perret-Liaudet A, Höftberger R, Liberski PP, Budka H, Sikorska B (2016). Tau Pathology in Creutzfeldt-Jakob Disease Revisited. Brain Pathol.

[37] Kovacs GG, Ferrer I, Grinberg LT, Alafuzoff I, Attems J, Budka H, Cairns NJ, Crary JF, Duyckaerts C, Ghetti B, Halliday GM, Ironside JW, Love S, Mackenzie IR, Munoz DG, Murray ME, Nelson PT, Takahashi H, Trojanowski JQ, Ansorge O, Arzberger T, Baborie A, Beach TG, Bieniek KF, Bigio EH, Bodi I, Dugger BN, Feany M, Gelpi E, Gentleman SM, Giaccone G, Hatanpaa KJ, Heale R, Hof PR, Hofer M, Hortobágyi T, Jellinger K, Jicha GA, Ince P, Kofler J, Kövari E, Kril JJ, Mann DM, Matej R, McKee AC, McLean C, Milenkovic I, Montine TJ, Murayama S, Lee EB, Rahimi J, Rodriguez RD, Rozemüller A, Schneider JA, Schultz C, Seeley W, Seilhean D, Smith C, Tagliavini F, Takao M, Thal DR, Toledo JB, Tolnay M, Troncoso JC, Vinters HV, Weis S, Wharton SB, White CL, Wisniewski T, Woulfe JM, Yamada M, Dickson DW (2016). Aging-related tau astrogliopathy (ARTAG): harmonized evaluation strategy. Acta Neuropathol.

[38] Ladogana A, Sanchez-Juan P, Mitrová E, Green A, Cuadrado-Corrales N, Sánchez-Valle R, Koscova S, Aguzzi A, Sklaviadis T, Kulczycki J, Gawinecka J, Saiz A, Calero M, van Duijn CM, Pocchiari M, Knight R, Zerr I (2009). Cerebrospinal fluid biomarkers in human genetic transmissible spongiform encephalopathies. J Neurol.

[39] Lewczuk P, Matzen A, Blennow K, Parnetti L, Molinuevo JL, Eusebi P, Kornhuber J, Morris JC, Fagan AM (2017). Cerebrospinal Fluid Aβ42/40 Corresponds Better than Aβ42 to Amyloid PET in Alzheimer’s Disease. J Alzheimers Dis.

[40] Mattsson N, Andreasson U, Persson S, Carrillo MC, Collins S, Chalbot S, Cutler N, Dufour-Rainfray D, Fagan AM, Heegaard NH, Hsiung GY, Hyman B, Iqbal K, Kaeser SA, Lachno DR, Lleó A, Lewczuk P, Molinuevo JL, Parchi P, Regeniter A, Rissman RA, Rosenmann H, Sancesario G, Schröder J, Shaw LM, Teunissen CE, Trojanowski JQ, Vanderstichele H, Vandijck M, Verbeek MM, Zetterberg H, Blennow K, Alzheimer’s Association QC Program Work Group (2013). CSF biomarker variability in the Alzheimer’s Association quality control program. Alzheimers Dement.

[41] McGuire LI, Peden AH, Orrú CD, Wilham JM, Appleford NE, Mallinson G, Andrews M, Head MW, Caughey B, Will RG, Knight RS, Green AJ (2012). Real time quaking-induced conversion analysis of cerebrospinal fluid in sporadic Creutzfeldt–Jakob disease. Ann Neurol.

[42] McGuire LI, Poleggi A, Poggiolini I, Suardi S, Grznarova K, Shi S, de Vil B, Sarros S, Satoh K, Cheng K, Cramm M, Fairfoul G, Schmitz M, Zerr I, Cras P, Equestre M, Tagliavini F, Atarashi R, Knox D, Collins S, Haïk S, Parchi P, Pocchiari M, Green A (2016). Cerebrospinal fluid real-time quaking-induced conversion is a robust and reliable test for sporadic creutzfeldt–jakob disease: An international study. Ann Neurol.

[43] McKhann GM, Knopman DS, Chertkow H, Hyman BT, Jack Jr CR, Kawas CH, Klunk WE, Koroshetz WJ, Manly JJ, Mayeux R, Mohs RC, Morris JC, Rossor MN, Scheltens P, Carrillo MC, Thies B, Weintraub S, Phelps CH (2011). The diagnosis of dementia due to Alzheimer’s disease: recommendations from the National Institute on Aging-Alzheimer’s Association workgroups on diagnostic guidelines for Alzheimer’s disease. Alzheimers Dement.

[44] Muayqil T, Gronseth G, Camicioli R (2012). Evidence-based guideline: diagnostic accuracy of CSF 14-3-3 protein in sporadic Creutzfeldt-Jakob disease: report of the guideline development subcommittee of the American Academy of Neurology. Neurology.

[45] Orrú CD, Bongianni M, Tonoli G, Ferrari S, Hughson AG, Groveman BR, Fiorini M, Pocchiari M, Monaco S, Caughey B, Zanusso G (2014). A test for Creutzfeldt-Jakob disease using nasal brushings. N Engl J Med.

[46] Orrú CD, Groveman BR, Hughson AG, Zanusso G, Coulthart MB, Caughey B (2015). Rapid and sensitive RT-QuIC detection of human Creutzfeldt-Jakob disease using cerebrospinal fluid. MBio.

[47] Otto M, Esselmann H, Schulz-Shaeffer W, Neumann M, Schröter A, Ratzka P, Cepek L, Zerr I, Steinacker P, Windl O, Kornhuber J, Kretzschmar HA, Poser S, Wiltfang J (2000). Decreased beta-amyloid1-42 in cerebrospinal fluid of patients with Creutzfeldt-Jakob disease. Neurology.

[48] Otto M, Wiltfang J, Cepek L, Neumann M, Mollenhauer B, Steinacker P, Ciesielczyk B, Schulz-Schaeffer W, Kretzschmar HA, Poser S (2002). Tau protein and 14-3-3 protein in the differential diagnosis of Creutzfeldt-Jakob disease. Neurology.

[49] Parchi P, Castellani R, Capellari S, Ghetti B, Young K, Chen SG, Farlow M, Dickson DW, Sima AA, Trojanowski JQ, Petersen RB, Gambetti P (1996). Molecular basis of phenotypic variability in sporadic Creutzfeldt-Jakob disease. Ann Neurol.

[50] Parchi P, Giese A, Capellari S, Brown P, Schulz-Schaeffer W, Windl O, Zerr I, Budka H, Kopp N, Piccardo P, Poser S, Rojiani A, Streichemberger N, Julien J, Vital C, Ghetti B, Gambetti P, Kretzschmar H (1999). Classification of sporadic Creutzfeldt-Jakob disease based on molecular and phenotypic analysis of 300 subjects. Ann Neurol.

[51] Parchi P, Notari S, Weber P, Schimmel H, Budka H, Ferrer I, Haik S, Hauw JJ, Head MW, Ironside JW, Limido L, Rodriguez A, Ströbel T, Tagliavini F, Kretzschmar HA (2009). Inter-laboratory assessment of PrPSc typing in Creutzfeldt-Jakob disease: a Western blot study within the NeuroPrion Consortium. Brain Pathol.

[52] Parchi P, Strammiello R, Notari S, Giese A, Langeveld JP, Ladogana A, Zerr I, Roncaroli F, Cras P, Ghetti B, Pocchiari M, Kretzschmar H, Capellari S (2009). Incidence and spectrum of sporadic Creutzfeldt-Jakob disease variants with mixed phenotype and co-occurrence of PrPSc types: an updated classification. Acta Neuropathol.

[53] Parchi P, Strammiello R, Giese A, Kretzschmar H (2011). Phenotypic variability of sporadic human prion disease and its molecular basis: past, present, and future. Acta Neuropathol.

[54] Parchi P, de Boni L, Saverioni D, Cohen ML, Ferrer I, Gambetti P, Gelpi E, Giaccone G, Hauw JJ, Höftberger R, Ironside JW, Jansen C, Kovacs GG, Rozemuller A, Seilhean D, Tagliavini F, Giese A, Kretzschmar HA (2012). Consensus classification of human prion disease histotypes allows reliable identification of molecular subtypes: an inter-rater study among surveillance centres in Europe and USA. Acta Neuropathol.

[55] Park JH, Choi YG, Lee YJ, Park SJ, Choi HS, Choi KC, Choi EK, Kim YS (2016). Real-time quaking-induced conversion analysis for the diagnosis of sporadic Creutzfeldt-Jakob disease in Korea. J Clin Neurol.

[56] Plate A, Benninghoff J, Jansen GH, Wlasich E, Eigenbrod S, Drzezga A, Jansen NL, Kretzschmar HA, Bötzel K, Rujescu D, Danek A (2013). Atypical parkinsonism due to a D202N Gerstmann-Sträussler-Scheinker prion protein mutation: first in vivo diagnosed case. Mov Disord.

[57] Prusiner SB (1998). Prions. Proc Natl Acad Sci USA.

[58] Puoti G, Zou W, Kong Q, Tagliavini F, Parchi P, Gambetti P (2009). Tau protein in a novel prion disease with GSS features. J Neuropath Exp Neurol.

[59] Reiniger L, Lukic A, Linehan J, Rudge P, Collinge J, Mead S, Brandner S (2011). Tau, prions and Aβ: the triad of neurodegeneration. Acta Neuropathol.

[60] Riemenschneider M, Wagenpfeil S, Vanderstichele H, Otto M, Wiltfang J, Kretzschmar H, Vanmechelen E, Förstl H, Kurz A (2003). Phospho-tau/total tau ratio in cerebrospinal fluid discriminates Creutzfeldt-Jakob disease from other dementias. Mol Psychiatry.

[61] Rosenbloom MH, Atri A (2011). The evaluation of rapidly progressive dementia. Neurologist.

[62] Sala I, Marquié M, Sánchez-Saudinós MB, Sánchez-Valle R, Alcolea D, Gómez-Ansón B, Gómez-Isla T, Blesa R, Lleó A (2012). Rapidly progressive dementia: experience in a tertiary care medical center. Alzheimer Dis Assoc Disord.

[63] Sanchez-Juan P, Green A, Ladogana A, Cuadrado-Corrales N, Sáanchez-Valle R, Mitrováa E, Stoeck K, Sklaviadis T, Kulczycki J, Hess K, Bodemer M, Slivarichová D, Saiz A, Calero M, Ingrosso L, Knight R, Janssens AC, van Duijn CM, Zerr I (2006). CSF tests in the differential diagnosis of Creutzfeldt-Jakob disease. Neurology.

[64] Sano K, Satoh K, Atarashi R, Takashima H, Iwasaki Y, Yoshida M, Sanjo N, Murai H, Mizusawa H, Schmitz M, Zerr I, Kim YS, Nishida N (2013). Early detection of abnormal prion protein in genetic human prion diseases now possible using real-time QUIC assay. PLoS One.

[65] Seppälä TT, Nerg O, Koivisto AM, Rummukainen J, Puli L, Zetterberg H, Pyykkö OT, Helisalmi S, Alafuzoff I, Hiltunen M, Jääskeläinen JE, Rinne J, Soininen H, Leinonen V, Herukka SK (2012). CSF biomarkers for Alzheimer disease correlate with cortical brain biopsy findings. Neurology.

[66] Sikorska B, Liberski PP, Sobów T, Budka H, Ironside JW (2009). Ultrastructural study of florid plaques in variant Creutzfeldt-Jakob disease: a comparison with amyloid plaques in kuru, sporadic Creutzfeldt-Jakob disease and Gerstmann-Sträussler-Scheinker disease. Neuropathol Appl Neurobiol.

[67] Skillbäck T, Rosén C, Asztely F, Mattsson N, Blennow K, Zetterberg H (2014). Diagnostic performance of cerebrospinal fluid total tau and phosphorylated tau in Creutzfeldt-Jakob disease: results from the Swedish Mortality Registry. JAMA Neurol.

[68] Stoeck K, Sanchez-Juan P, Gawinecka J, Green A, Ladogana A, Pocchiari M, Sanchez-Valle R, Mitrova E, Sklaviadis T, Kulczycki J, Slivarichova D, Saiz A, Calero M, Knight R, Aguzzi A, Laplanche JL, Peoc’h K, Schelzke G, Karch A, van Duijn CM, Zerr I (2012). Cerebrospinal fluid biomarker supported diagnosis of Creutzfeldt-Jakob disease and rapid dementias: a longitudinal multicentre study over 10 years. Brain.

[69] Strozyk D, Blennow K, White LR, Launer LJ (2003). CSF Abeta 42 levels correlate with amyloid-neuropathology in a population-based autopsy study. Neurology.

[70] Tapiola T, Alafuzoff I, Herukka SK, Parkkinen L, Hartikainen P, Soininen H, Pirttilä T (2009). Cerebrospinal fluid β-amyloid 42 and tau proteins as biomarkers of Alzheimer-type pathologic changes in the brain. Arch Neurol.

[71] Van Everbroeck B, Green AJ, Pals P, Martin JJ, Cras P (1999). Decreased Levels of Amyloid-beta 1-42 in Cerebrospinal Fluid of Creutzfeldt-Jakob Disease Patients. J Alzheimers Dis.

[72] Van Everbroeck B, Quoilin S, Boons J, Martin JJ, Cras P (2003). A prospective study of CSF markers in 250 patients with possible Creutzfeldt–Jakob disease. J Neurol Neurosurg Psychiatry.

[73] Varges D, Jung K, Gawinecka J, Heinemann U, Schmitz M, von Ahsen N, Krasnianski A, Armstrong VW, Zerr I (2011). Amyloid-β 1-42 levels are modified by apolipoprotein E ε4 in Creutzfeldt-Jakob disease in a similar manner as in Alzheimer’s disease. J Alzheimers Dis.

[74] Will RG (2003). Acquired prion disease: iatrogenic CJD, variant CJD, kuru. Br Med Bull.

[75] Wiltfang J, Esselmann H, Smirnov A, Bibl M, Cepek L, Steinacker P, Mollenhauer B, Buerger K, Hampel H, Paul S, Neumann M, Maler M, Zerr I, Kornhuber J, Kretzschmar HA, Poser S, Otto M (2003). Beta-amyloid peptides in cerebrospinal fluid of patients with Creutzfeldt-Jakob disease. Ann Neurol.

[76] Wiltfang J, Esselmann H, Bibl M, Hüll M, Hampel H, Kessler H, Frölich L, Schröder J, Peters O, Jessen F, Luckhaus C, Perneczky R, Jahn H, Fiszer M, Maler JM, Zimmermann R, Bruckmoser R, Kornhuber J, Lewczuk P (2007). Amyloid beta peptide ratio 42/40 but not A beta 42 correlates with phospho-Tau in patients with low- and high-CSF A beta 40 load. J Neurochem.

[77] Zanusso G, Fiorini M, Ferrari S, Gajofatto A, Cagnin A, Galassi A, Richelli S, Monaco S (2011). Cerebrospinal fluid markers in sporadic Creutzfeldt-Jakob disease. Int J Mol Sci.

[78] Zanusso G, Monaco S, Pocchiari M, Caughey B (2016). Advanced tests for early and accurate diagnosis of Creutzfeldt-Jakob disease. Nat Rev Neurol.

[79] Zerr I, Pocchiari M, Collins S, Brandel JP, de Cuesta Pedro J, Knight RS, Bernheimer H, Cardone F, Delasnerie-Lauprêtre N, Corrales NC, Ladogana A, Bodemer M, Fletcher A, Awan T, Ruiz Bremón A, Budka H, Laplanche JL, Will RG, Poser S (2000). Analysis of EEG and CSF 14-3-3 proteins as aids to the diagnosis of Creutzfeldt–Jakob disease. Neurology.

[80] Zerr I, Kallenberg K, Summers DM, Romero C, Taratuto A, Heinemann U, Breithaupt M, Varges D, Meissner B, Ladogana A, Schuur M, Haik S, Collins SJ, Jansen GH, Stokin GB, Pimentel J, Hewer E, Collie D, Smith P, Roberts H, Brandel JP, van Duijn C, Pocchiari M, Begue C, Cras P, Will RG, Sanchez-Juan P (2009). Updated clinical diagnostic criteria for sporadic Creutzfeldt-Jakob disease. Brain.

[81] Zou WQ, Puoti G, Xiao X, Yuan J, Qing L, Cali I, Shimoji M, Langeveld JP, Castellani R, Notari S, Crain B, Schmidt RE, Geschwind M, Dearmond SJ, Cairns NJ, Dickson D, Honig L, Torres JM, Mastrianni J, Capellari S, Giaccone G, Belay ED, Schonberger LB, Cohen M, Perry G, Kong Q, Parchi P, Tagliavini F, Gambetti P (2010). Variably protease-sensitive prionopathy: a new sporadic disease of the prion protein. Ann Neurol.

