# Supplementary material for: Prion-specific and surrogate CSF biomarkers in Creutzfeldt-Jakob disease: diagnostic accuracy in relation to molecular subtypes and analysis of neuropathological correlates of p-tau and Aβ42 levels
Source: Acta Neuropathol. 2017 Feb 15;133(4):559–78. doi: 10.1007/s00401-017-1683-0 (PMC5348556; doi:10.1007/s00401-017-1683-0)
Supplement: Supplementary file 1 — Supplementary material 1 (DOCX 42 kb) [file 401_2017_1683_MOESM1_ESM.docx]

**Supplementary materials**

**Table 1. Number of samples for each group of disorders/subtypes used for the analyses of CSF p-tau, t-tau/p-tau ratio and Aβ42***

| **Diagnostic categories** | **CSF (n)** | |
| --- | --- | --- |
|  | **p-tau and t-tau/p-tau** | **Aβ42*** |
| Definite sCJD (all subtypes) | 174 | 116 |
| Probable sCJD | 63 | 19 |
| Possible sCJD | 13 | 0 |
| Definite VPSPr | 1 | 1 |
| *sCJD codon 129* |  |  |
| MM | 146 | 96 |
| MV | 62 | 39 |
| VV | 43 | 29 |
| *Major sCJD subtypes* **^§^** |  |  |
| MM(V)1 | 103 | 72 |
| VV2 | 27 | 21 |
| MV2K | 24 | 15 |
| MM2C | 10 | 3 |
| Genetic CJD (all haplotypes) | 43 | 28 |
| *Major gCJD haplotypes ^§^* |  |  |
| E200K-129M | 15 | 12 |
| V210I-129M | 20 | 13 |
| All CJD | 294 | 164 |
| Definite CJD (all prions) | 218 | 144 |
| All non-CJD | 311 | 121 |
| “Definite” non-CJD | 142 | 25 |

*Only the number of samples with a storage time up to 5 years are included **^§^**The number of samples for the rare sCJD subtypes (MM2T and VV1) are not listed; ***^§^*** The number of samples for the rare gCJD haplotypes are not listed.

**Table 2. Analysis of the effect of storage time on results of p-tau, Aβ42, and RT-QuIC assays**

| **Storage time (years)** | **p-tau (pg/ml)**  **Median (IQR)** | **n** | **Aβ42 (pg/ml)**  **Median (IQR)** | **n** | **RT-QuIC**  **% positive** | **n** |
| --- | --- | --- | --- | --- | --- | --- |
| <1  ≥1 and ≤5  >5 | 49 (37-67)  41 (33-58)  43 (34-57) | 45  81  22 | 587 (383-790)  467 (360-703)  358 (226–567) | 51  113  44 | 87.3  81.0  82.0 | 71  163  89 |

In the group of definite and probable CJD there was an effect of storage time on Aβ42 levels. In detail, the group with storage time <1 year and the group with storage time ≥1 year and ≤ 5 years did not differ in Aβ42 levels (p= 0.228). Otherwise, in the group with storage time >5 years we observed a significant reduction of Aβ42 levels in comparison to the group with storage time ≥1 year and ≤ 5 years (p=0.012) and the group with storage time <1 year (p=0.003). In the group of definite and probable CJD there was no effect of storage time on RT-QuIC results (p=0.420). Regarding p-tau levels the analysis on definite MM(V)1 and probable MM showed no effect of storage time on p-tau levels (p=0.134).

**Table 3. Results of 14-3-3 and t-tau assays in neuropathologically verified non-CJD according to diagnostic category**

| **Diagnostic categories** | **14-3-3 positive/total** | **t-tau positive/total** |
| --- | --- | --- |
| Alzheimer’s disease | 4/15 | 4/15 |
| Lewy body dementia | 3/13 | 4/13 |
| Tauopathy NFT only | 0/1 | 0/1 |
| Mixed dementia | 0/1 | 0/1 |
| Vascular dementia | 7/10 | 6/10 |
| Stroke | 1/1 | 0/1 |
| CNS malignancy | 6/8 | 4/8 |
| Non-specific encephalitis | 6/8 | 5/8 |
| Autoimmune encephalitis | 3/4 | 3/4 |
| EBV encephalitis | 1/1 | 1/1 |
| PML | 1/1 | 1/1 |
| Wernicke encephalopathy | 2/3 | 1/3 |
| Metabolic encephalopathy | 0/1 | 0/1 |
| Ceroidolipofuscinosis | 0/1 | 0/1 |
| Cause undefined | 3/13 | 2/13 |
| **Total** | **37/81** | **31/81** |

**Table 4. N° of non-CJD samples used for the p-tau assay according to diagnostic category.**

| **Diagnostic categories of non-CJD patients** | **p-tau evaluated/total** |
| --- | --- |
| Alzheimer’s disease | 85/101 |
| Lewy body dementia | 27/72 |
| Frontotemporal dementia | 10/40 |
| Other NDG diseases | 8/51 |
| Mixed dementia | 12/35 |
| Vascular dementia | 29/85 |
| CNS malignancy | 17/18 |
| Inflammatory condition / encephalitis / paraneoplastic sindrome | 64/130 |
| Toxic/ Metabolic encephalopathy | 6/20 |
| Psychiatric | 0/8 |
| Other diseases | 2/13 |
| Cause unknown | 51/130 |
| Total | 311/703 |

**Table 5. Assessment of neuronal and glial tau pathology in major CJD subtypes (n=158).**

| **Molecular subtype** | ***N°* of cases** | **Age at death**  **(mean±SD)** | **Braak stages** | | | **AD-tau score**  **(mean±SD)** | **ARTAG**** |
| --- | --- | --- | --- | --- | --- | --- | --- |
|  |  |  | 0/+ | 1-2 | 3-4* |  |  |
| MM(V)1 | 109 | 68±9 | 58 (53%) | 37 (34%) | 14 (13%) | 7.7±7.4 | 5 (4.5%) |
| VV2 | 25 | 69±9 | 15 (60%) | 7 (32%) | 3 (12%) | 5.3±7.6 | 2 (8%) |
| MV2K | 16 | 66±9 | 12 (75%) | 4 (25%) | 0 (0%) | 4.6±4.4 | 0 (0%) |
| MM2C | 8 | 64±11 | 6 (75%) | 1 (12.5%) | 1 (12.5%) | 2.7±3.4 | 0 (0%) |

* Braak stage V was detected in a single MM1 brain. **Assessed on sections required for Braak staging (see materials and methods)

**Table 6. Demographic characteristics of the studied CJD population.**

| **Diagnostic categories** | **n** | **Age (years)** | **Female (%)** | **Time from onset to LP** | **Disease Duration** |
| --- | --- | --- | --- | --- | --- |
| Definite sCJD | 186 | 68 (61-75) | 53.2 | 2 (1-4) | 5 (2.5-9) |
| MM(V)1 | 111 | 69 (62-77) | 45.9 | 1.3 (1-2) | 3 (2-5) |
| VV2 | 29 | 67 (60.5-77) | 62.1 | 3.5 (3-4.5) | 6 (5.3-7.8) |
| MV2K | 26 | 62.5 (58.2-68) | 69.2 | 6.3 (3.9-10) | 16 (12-21.3) |
| MM2C | 10 | 67 (55.5-73) | 60 | 9 (4.6-11.8) | 12.5 (6-34.1) |
| Probable sCJD | 97 | 70 (63-74) | 60.8 | 2 (1-5) | 7 (3-15) |
| Possible sCJD | 29 | 69 (66.5-73.5) | 48.3 | 3 (1.5-6) | 4.5 (4-14) |
| Definite VPSPr | 1 | 72 | 100 | 3.0 | 36.0 |
| *Codon 129* |  |  |  |  |  |
| MM | 195 | 69 (62-76) | 55.9 | 1.5 (1-2.2) | 3.5 (2-7) |
| MV | 72 | 68 (62-72.8) | 56.9 | 5.8 (2.5-9.5) | 14.5 (9.5-19) |
| VV | 46 | 69 (60.8-75.3) | 50 | 3 (2.9-4.5) | 6 (5-8) |
| Genetic CJD | 46 | 64 (55-70.5) | 60.9 | 1.6 (1-3.5) | 4 (3-10) |
| *Maior mutation type* |  |  |  |  |  |
| E200K-129M | 16 | 59.5(49.8-72.8) | 50 | 2 (1-4.4) | 4 (3-15) |
| V210I-129M | 21 | 64 (58.5-72) | 66.7 | 1.5 (1-1.7) | 3 (1.9-5.6) |
| **All CJD** | **359** | **68 (61-74)** | **56** | **2 (1-5)** | **5 (2.5-11)** |

**Table 7. Analysis of the effect of sex and age on results of p-tau, Aβ42, and RT-QuIC assays**

|  | Aβ42 (pg/ml)  Median (IQR) | n | p-tau (pg/ml)  Median (IQR) | n | RT-QuIC  % positive | n |
| --- | --- | --- | --- | --- | --- | --- |
| Sex  Male  Female | 569 (380-757)  460 (353-687) | 80  84 | 49 (36-66)  51 (35-70) | 130  151 | 80.9  83.5 | 141  182 |
| Age (years)  <63    ≥63 and <72    ≥72 | 448 (353-699)  638 (414-774)  447(353-703) | 57  51  56 | 44 (34-64)  49 (37-67)  52 (40-72) | 95  93  92 | 78.2  82.9  85.3 | 101  105  116 |

In the group of definite and probable CJD patients, there was no effect of sex on Aβ42 (p=0.123), p-tau levels (p=0.496) and RT-QuIC results (p=0.533). In the same group, when stratifying p-tau, Aβ42, RT-QuIC results by age, there was no effect of age on Aβ42 (p=0.061), p-tau (p=0.115) levels and RT-QuIC results (p=0.384).

**Table 8. Analysis of the effect of LP “timing” and disease duration on results of t-tau, p-tau, Aβ42, and RT-QuIC assays**

|  | 14-3-3  % positive | n | t-tau (pg/ml)  Median (IQR) | n | p-tau (pg/ml)  Median (IQR) | n | Aβ42 (pg/ml)  Median (IQR) | n | RT QuIC  %  positive | n |
| --- | --- | --- | --- | --- | --- | --- | --- | --- | --- | --- |
| Time from onset to LP (months)  <1  ≥1 and <2  ≥2 | 85.0  92.7  97.2 | 20  55  36 | 6191 (3018-11275)  7586 (3443-11500)  6289 (3200-11705) | 20  55  36 | 41 (34-50)  47 (33-60)  43 (36-60) | 20  49  34 | 623 (281-741)  511 (407-705)  684 (378-759) | 12  39  21 | 100  86.5  85.7 | 20  52  35 |
| Disease duration  (months)  <2  ≥2 and <4  ≥4 | 88.9  96.2  90.2 | 18  52  41 | 8066 (3596-13150)  6774 (3151-11600)  6639 (3096-10388) | 18  52  41 | 50 (35-61)  45 (34-60)  43 (34-55) | 16  48  39 | 465 (310-645)  568 (348-718)  541 (411-759) | 11  36  25 | 88.2  90.0  87.5 | 17  50  40 |
| Time from LP to death (months)  <1  ≥1 and <2  ≥2 | 94.9  96.6  88.4 | 39  29  43 | 7813 (4911-12400)  6744 (3151-11600)  6696 (2890-9976) | 39  29  43 | 48 (35-64)  45 (34-60)  42 (34-53) | 34  27  42 | 477 (382-669)  707 (398-766)  505 (378-717) | 22  21  29 | 88.9  92.9  86 | 36  28  43 |

In the group of definite sporadic MM(V)1 there was no effect of time from clinical onset to LP on Aβ42 (p=0.691), t-tau (p=0.815), p-tau levels (p=0.707), 14-3-3 (p=0.238) and RT-QuIC results (p=0.210).

In the same group there was no effect of disease duration on Aβ42 (p=0.566), t-tau (p=0.636), p-tau levels (p=0.515), 14-3-3 (p=0.430) and RT-QuIC results (p=0.930).

In the same group there was no effect of time from LP to death on Aβ42 (p=0.256), t-tau (p=0.225), p-tau levels (p=0.248), 14-3-3 (p=0.346) and RT-QuIC results (p=0.673).
